# Supplementary material for: Developmental features and unique characteristics of peptide-specific PLZF+ innate-like T cells in mice
Source: Nat Commun. 2025 Jun 6;16:5274. doi: 10.1038/s41467-025-60617-4 (PMC12144119; doi:10.1038/s41467-025-60617-4)
Supplement: Supplementary file 1 — Supplementary Information [file 41467_2025_60617_MOESM1_ESM.pdf]

Developmental features and unique characteristics of peptide-specific PLZF^{+} innate-like T cells in mice

Ahmed Hassan, Nico Heise, Anja Schimrock, Stefanie Willenzon, Inga Ravens, Reinhold Förster, and Hristo Georgiev*

*Corresponding author: Hristo Georgiev (georgiev.hristo@mh-hannover.de)

This file contains:

Supplementary Figures 1-10

Supplementary Tables 1-4

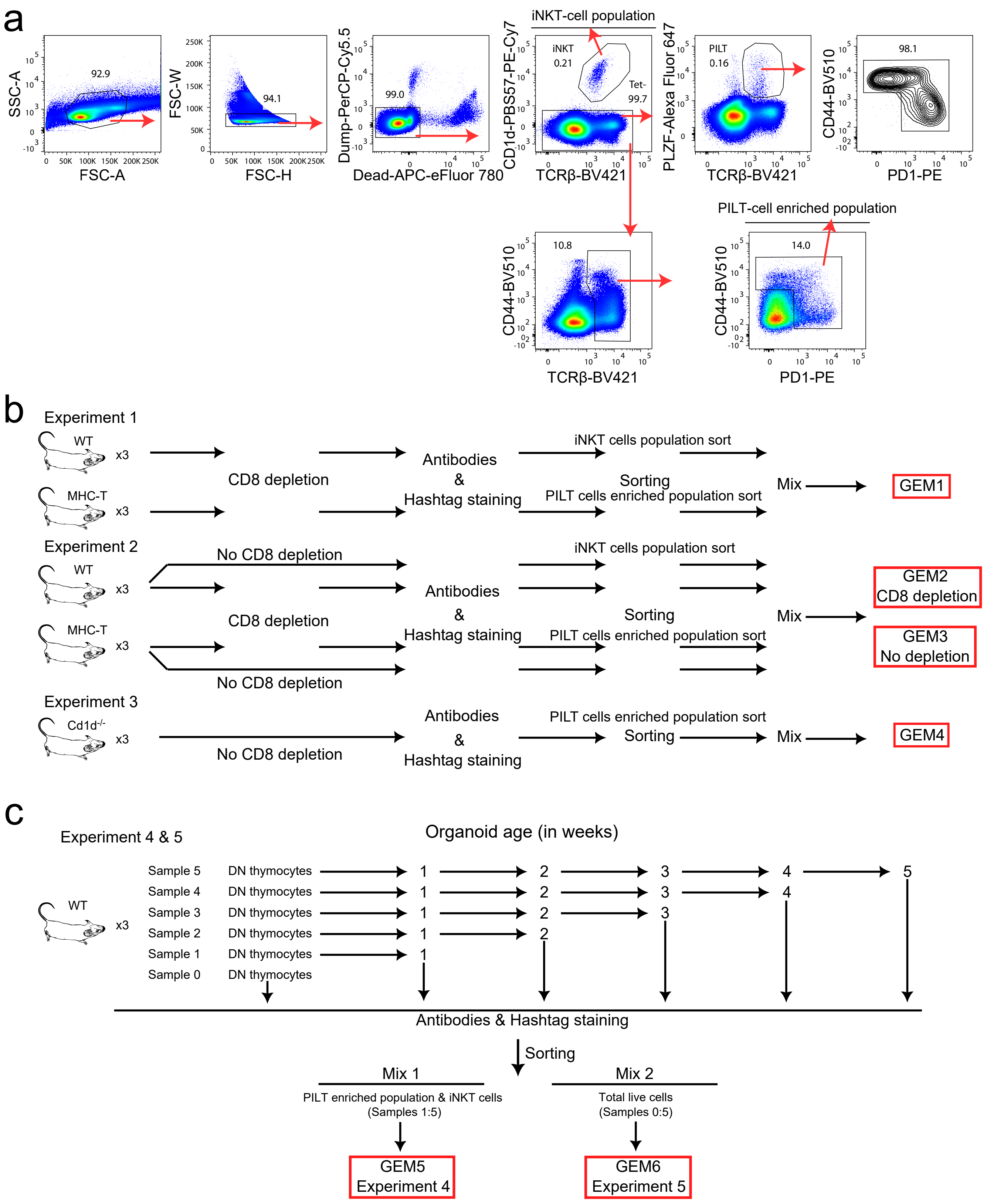

Supplementary Fig. 1 | Gating strategy and single-cell RNA sequencing experimental layout. **a**. The full gating strategy used for flow cytometry analysis allowing for the identification of iNKT, PILT and PILT-cell enriched cell populations. The same gating strategy was applied for sorting PILT-cell enriched cell populations used for single-cell RNA sequencing experiments. Schematic drawing showing the experimental layouts used for single-cell RNA sequencing for thymic-derived samples in (**b**) and ATOC-derived samples in (**c**). Detailed information for each single-cell RNA sequencing experiment is provided in the Supplementary Table 4. GEM is an abbreviation for gel bead-in-emulsion.

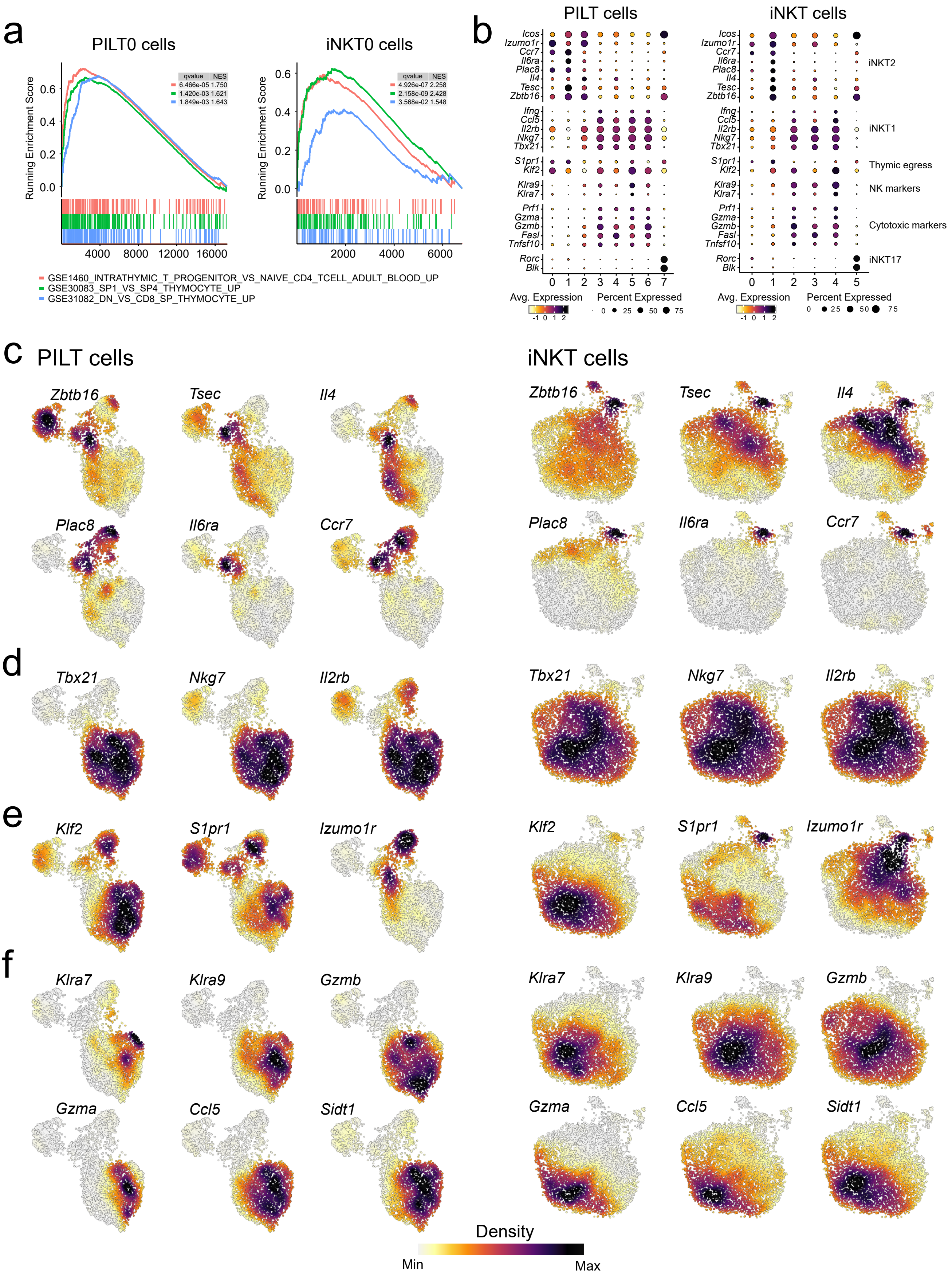

Supplementary Fig. 2 | Transcriptional profile of PILT and iNKT cells subsets.

a. Gene set enrichment analysis of PILT0 and iNKT0 subsets (from Fig. 2a and 2c) compared to the rest of the subsets using publicly available gene signatures of i. Intrathymic T cell progenitors vs adult blood naive CD4 T cells (red), ii. Early CD4 SP (SP1) vs late CD4 SP (SP4) (green) and, iii. DN vs CD8 SP (blue). **b**. Dot plots showing the scaled log normalised average expression of selected gene markers for each cluster for PILT cells (Fig. 2a) and iNKT cells (Fig. 2c). **c-f** Density plots for selected gene markers for PILT cells (Fig. 2a) and iNKT cells (Fig. 2c).

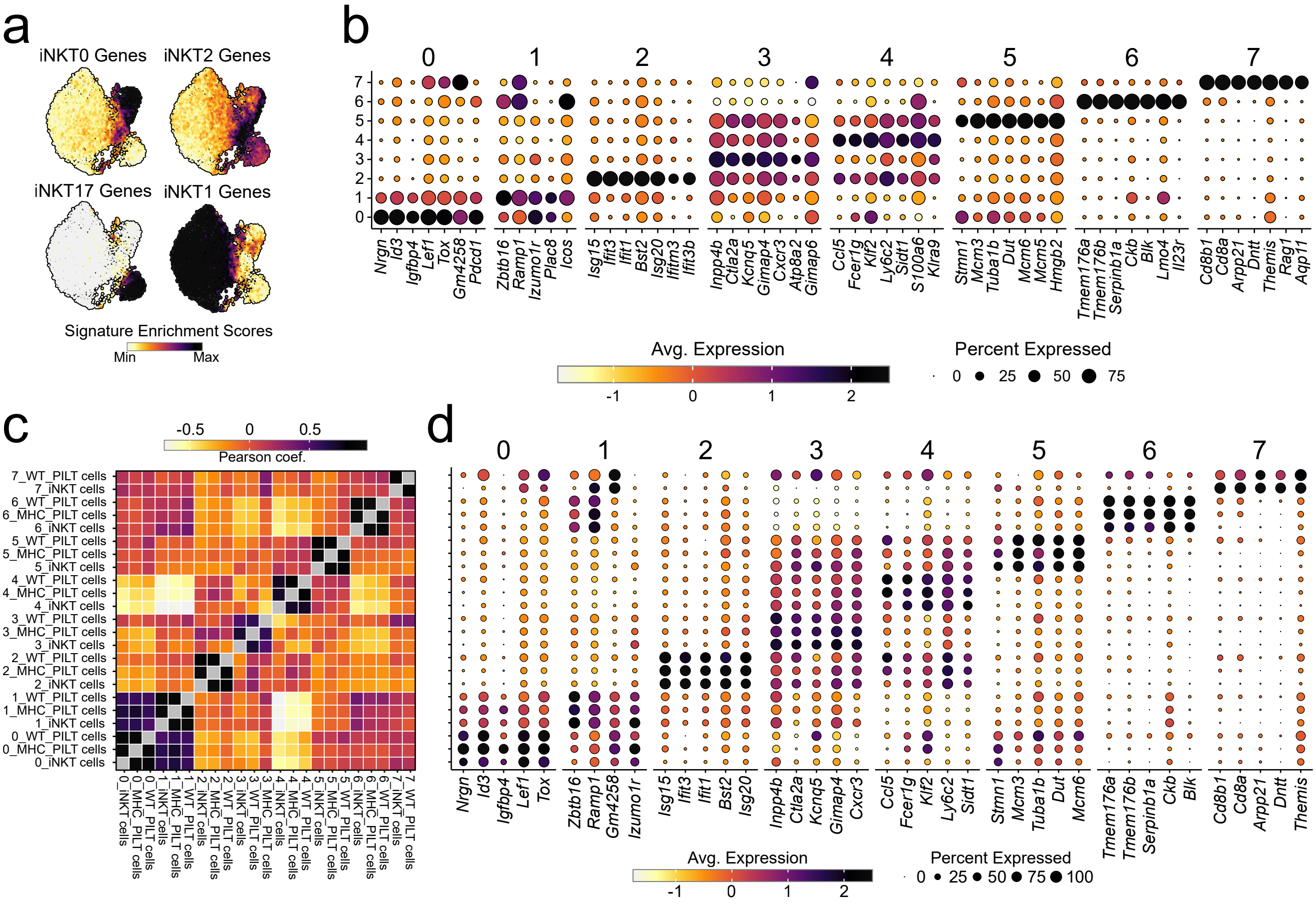

Supplementary Fig. 3 | Transcriptional signature of integrated PILT and iNKT cell dataset. **a**. Feature plot showing the k-nearest neighbours (KNN) smoothed UCell signature enrichment score for iNKT subsets signature gene markers (Supplementary Table 1) for cells in (Fig. 3a). **b**. Dot plot showing the scaled log normalised average expression of the top 7 differentially expressed gene markers for each cluster in (Fig. 3a). **c**. Pearson's correlation between iNKT, MHC-PILT and, WT-PILT cells in each cluster in (Fig. 3a) based on the top 10 differentially expressed gene markers for each cluster. **d**. Dot plot showing the scaled log normalised average expression of the top 5 differentially expressed gene markers for each cluster from (Fig. 3a) split by iNKT, MHC-PILT and, WT-PILT cells. WT-PILT is an abbreviation for derived from CD1d$^{-/-}$ mice; MHC-PILT for derived from T-MHC I mice peptide-specific PLZF+ innate-like T and iNKT for invariant natural killer T cells.

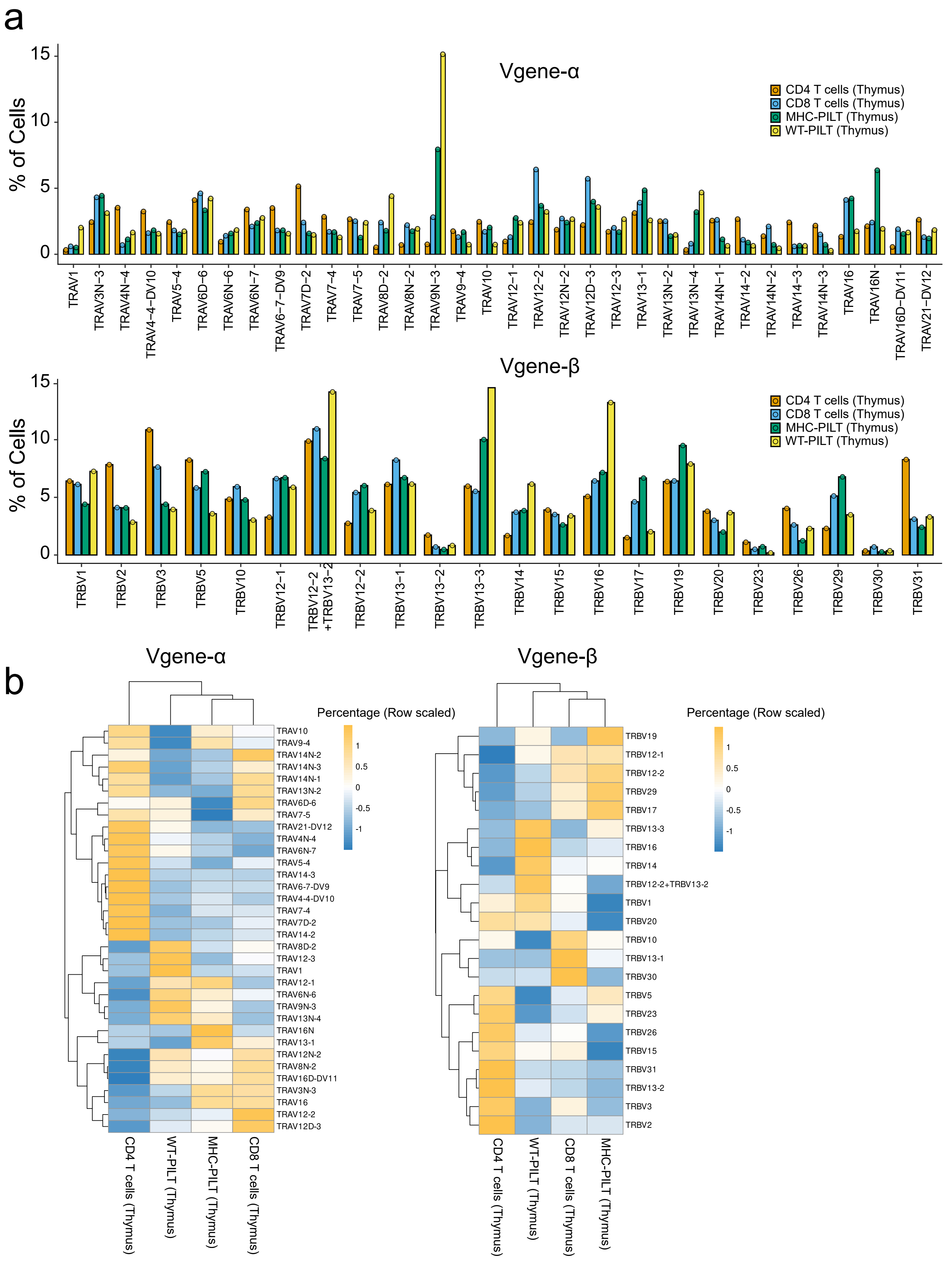

Supplementary Fig. 4 | Comparison of Vgene-α and Vgene-β usage between PILT cells and other T cells populations.

Bar plots in (**a**) and heat maps in (**b**) showing the Vgene-α and, Vgene-β usage for PILT cells, CD8 and CD4 T cells populations from thymus. WT-PILT is an abbreviation for derived from CD1d$^{-/-}$ mice and MHC-PILT for derived from T-MHC I mice peptide-specific PLZF+ innate-like T cells. Source data are provided as a Source Data file.

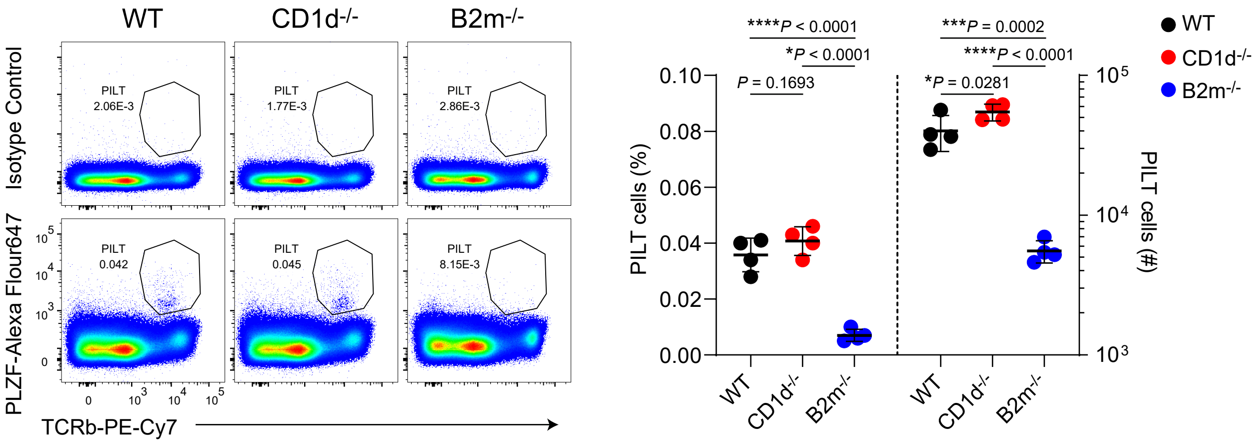

Supplementary Fig. 5 | Flow cytometry evaluation of PILT-cell frequencies and numbers in the thymus.

Representative flow cytometry plots (left) and a scatter plot (right) showing thymic PILT-cell frequency (plotted on the left axis) and number (plotted on the right axis) from WT, $CD1d^{-/-}$, and $B2m^{-/-}$ transgenic mice. Values are calculated by subtracting Isotype controls from the PLZF staining. PILT cells are gated as Live/$CD19^{-}F4/80^{-}CD1d\text{-}Tet^{-}MR1\text{-}Tet^{-}TCRb^{+}PLZF^{+}$. Each point represents one animal: n = 4 animals per group. Data are representative of two biologically independent experiments. Statistical significance was calculated using one-way ANOVA followed by Fisher's LSD multiple comparisons test; not significant ($P \geq 0.05$), *$P < 0.05$, ***$P < 0.001$ and ****$P < 0.0001$. Data are presented as mean values ±SD.

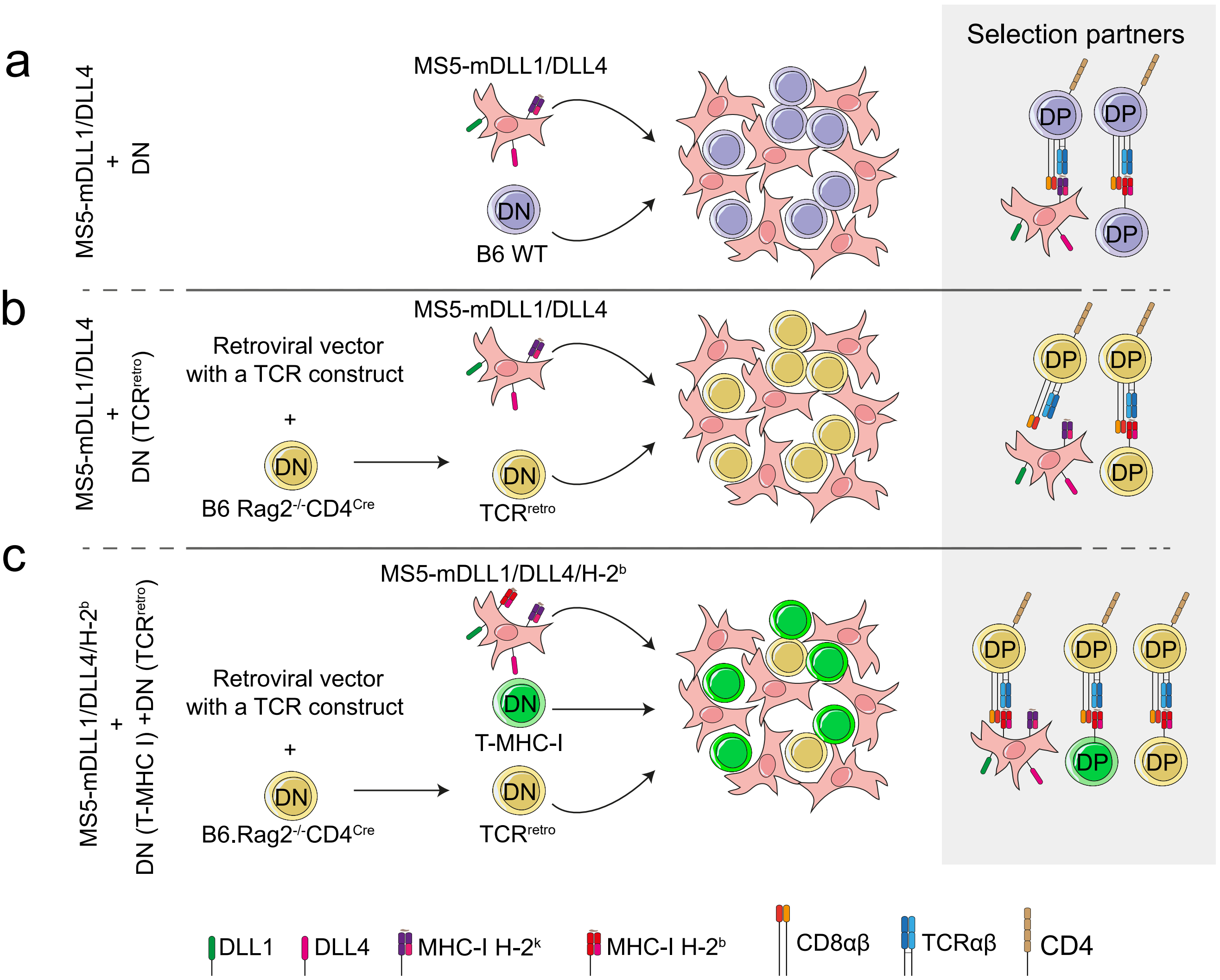

Supplementary Fig. 6 | Experimental design for ATOC experiments.

a-c. Schematic drawings showing the experimental setup in each ATOC condition. DN is an abbreviation for double negative and DP for double positive thymocyte-cell populations.

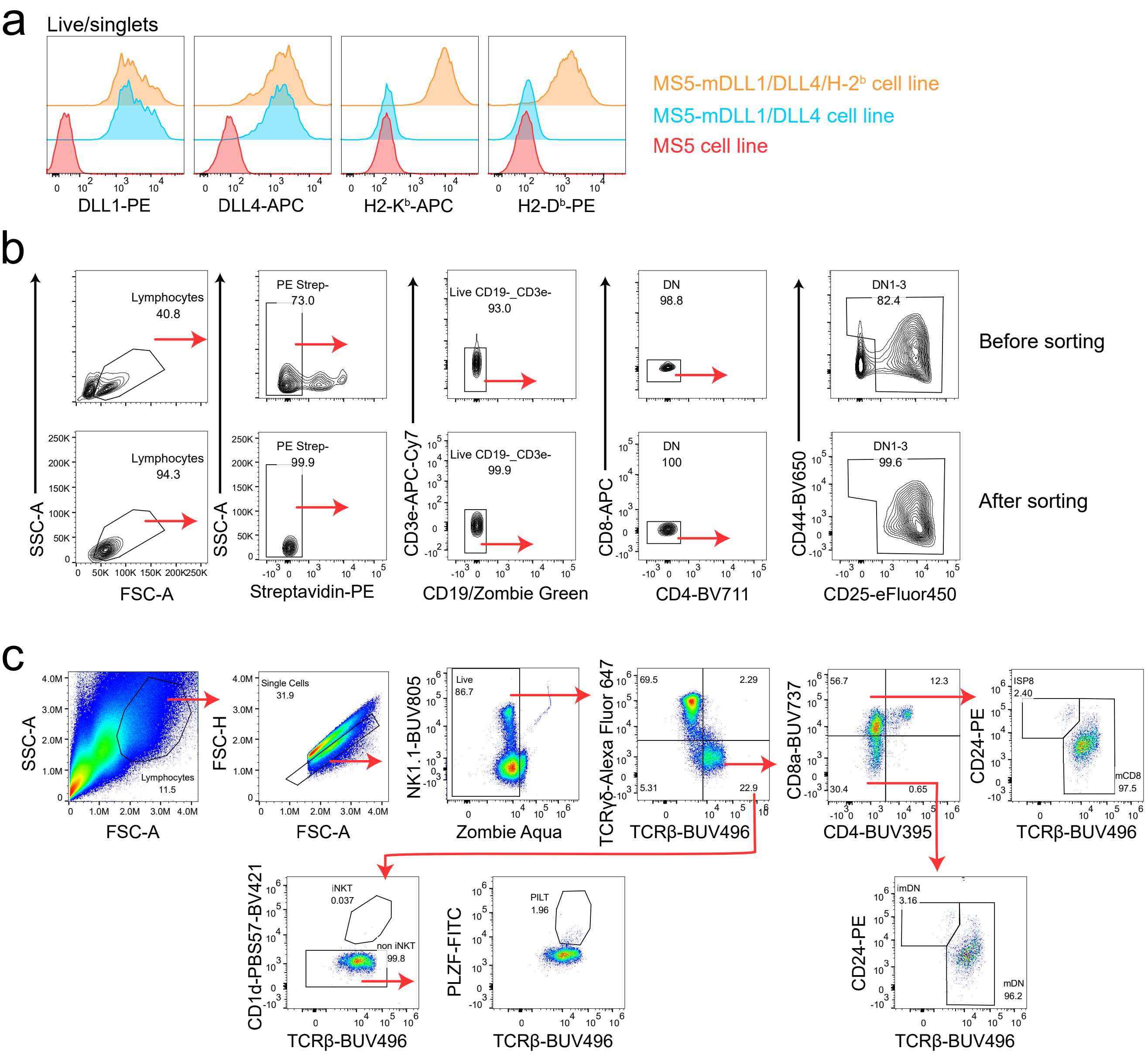

Supplementary Fig. 7 | Gating strategies applied for cells sorting and flow cytometry analysis of ATOCs.

a. Flow cytometry analysis showing the expression of mDLL1, mDLL4, H2-K^b and H2-D^b on different MS-5 cell lines. Full gating strategies applied for cell sorting in **b** and for flow cytometry analysis in **c** of the indicated cell populations developing in the ATOCs.

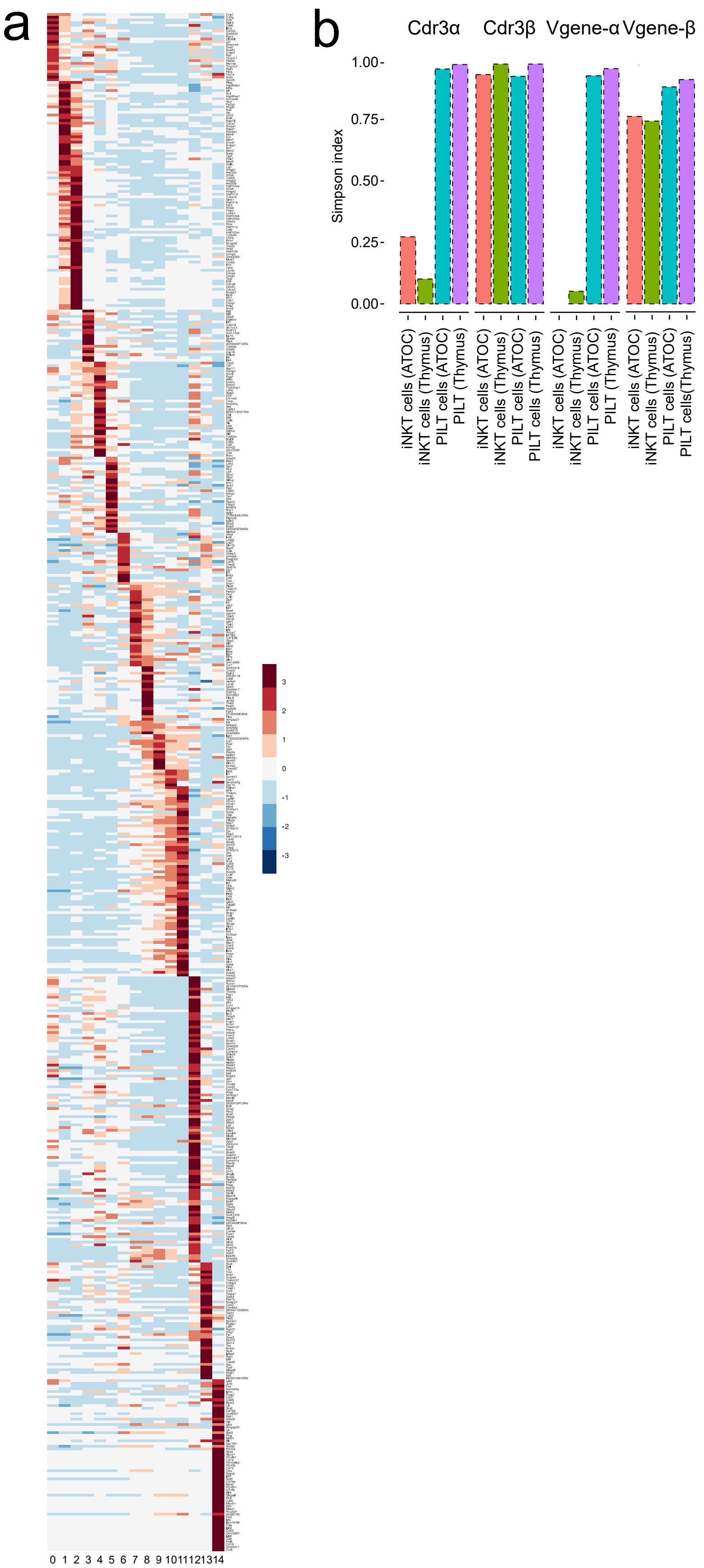

Supplementary Fig. 8 | Transcriptional profile of ATOC-generated cell populations over the course of 5 weeks.

a. A heatmap showing the aggregate normalised called expression of the top 100 expression of the top gene markers per cluster from (Fig. 6a) **b.** bar plot showing the Simpson diversity index score of Cdr3α, Cdr3β, Vgene-α and, Vgene-β usage for down sampled thymic and ATOC generated PILT and iNKT cells. PILT is an abbreviation for peptide-specific PLZF^{+} innate-like T cells and iNKT for invariant natural killer T cells. Source data are provided as a Source Data file.

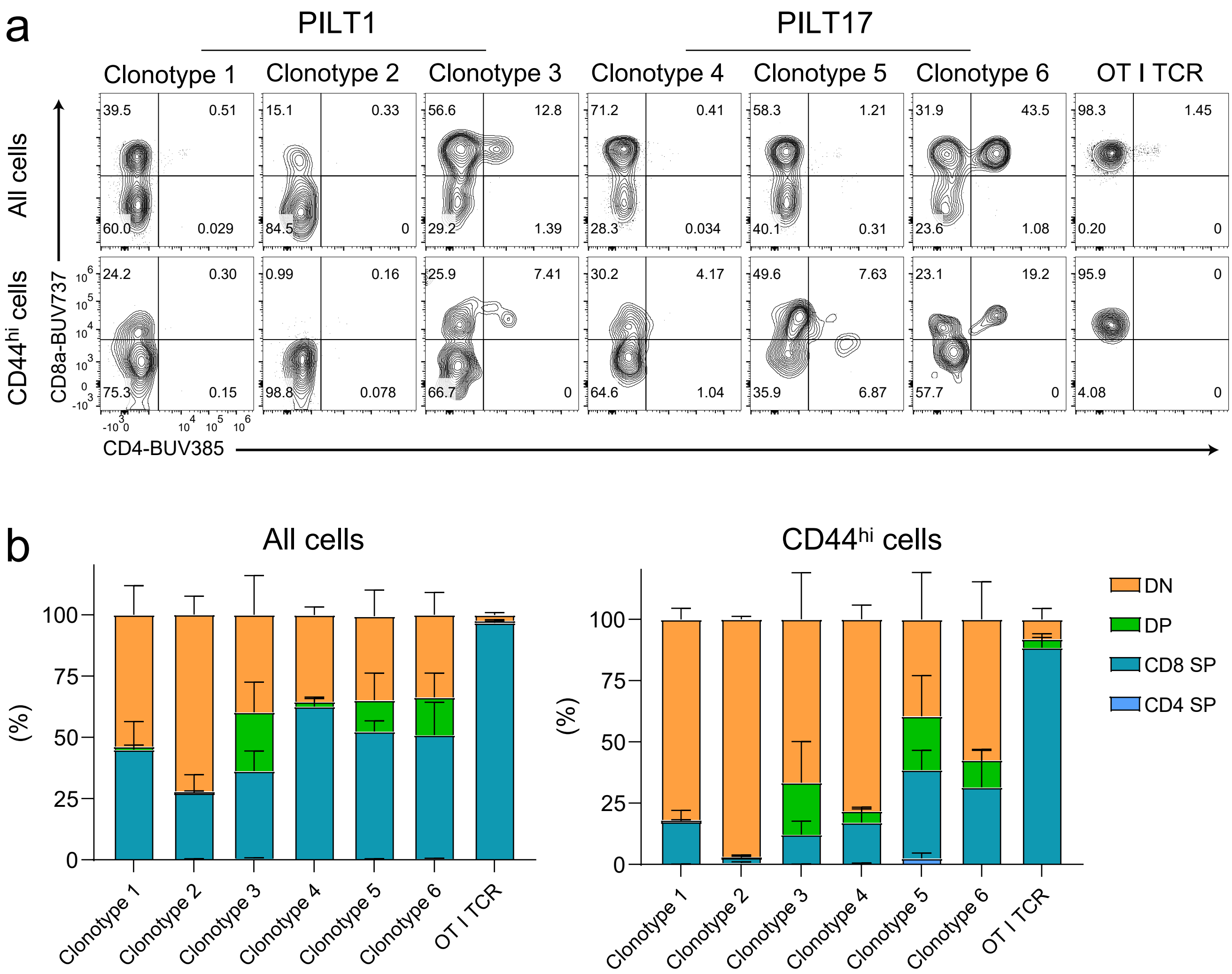

Supplementary Fig. 9 | T-cell receptor retrogenic PILT cells downregulate CD4 and CD8α co-receptor expression.

a. Representative flow cytometry plots showing CD4 and CD8 α expression on all Thy1.1$^+$ T cells (upper row) and Thy1.1$^+$CD44^{hi} T cells (lower row) generated in the T-cell receptor retrogenic ATOC system. **b.** Data quantification according to the gating strategy displayed in (**a**). DN is an abbreviation for double negative; DP for double positive, CD8 SP for CD8 single positive and CD4 SP for CD4 single positive thymocyte-cell populations. ATOCs were generated as depicted in Supplementary Fig. 5c. n=4 ATOCs per group. Data are representative of two biologically independent experiments. Data are presented as mean values ±SEM. Source data are provided as a Source Data file.

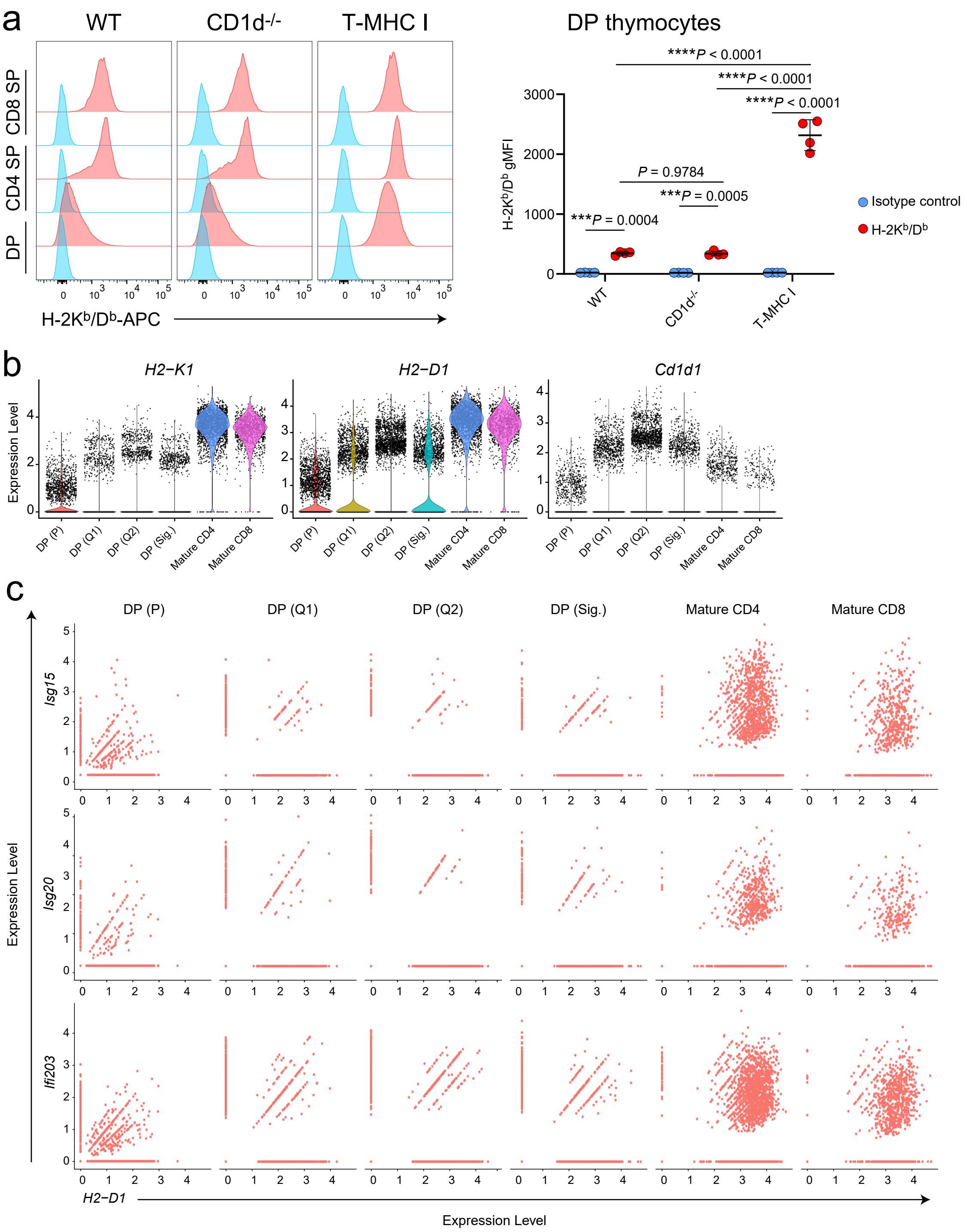

Supplementary Fig. 10 | MHC-I expression levels on WT DP thymocytes.

a. Representative flow cytometry plots (left) and a scatter plot (right) showing MHC I expression on double positive (DP); CD4 single positive (CD4 SP) and CD8 single positive (CD8 SP) thymocyte-cell populations from WT, CD1d$^{-/-}$ and T-MHC I transgenic mice. **b.** Violin plots showing *H2-K1*, *H2-D1* and *Cd1d1* normalized expression levels within DP (P), double positive proliferating; DP (Q1), DP quiescent 1; DP (Q2), DP quiescent 2; DP (Sig.), DP signaled; Mature CD4 and Mature CD8 thymocyte-cell populations as annotated by Steier *et al.* **c**. Feature scatter plots showing *H2-D1*, *Isg15*, *Isg20* and *Ifi203* co-expression levels within thymocyte-cell populations as annotated by Steier *et al.* Data shown in **b** and **c** were obtained from B6 WT scRNAseq dataset described in Steier *et al*. Each point represents one animal: n = 4 animals per group in **a**. Data are representative of two biologically independent experiments in **a**. Statistical significance was calculated using one-way ANOVA followed by Fisher's LSD multiple comparisons test in **a**; not significant ($P \geq 0.05$), ***$P < 0.001$, and ****$P < 0.0001$. Data are presented as mean values ±SD. Source data are provided as a Source Data file.

Supplementary Table 1: List of iNKT subset markers

	iNKT0	iNKT1	iNKT2	iNKT17
Markers	Itm2a	Klrb1c	Zbtb16	Rorc
	Ccr9	Ccl5	Il4	Sdc1
	Lef1	Xcl1	Gata3	Il23r
	Cd24a	Fcer1g	Il6ra	Il17rb
	Cd81	Ifng	Plac8	Il1r1
	Egr1	Gzma	Pdcd1	Ccr6
	Egr2	Gzmb	PD1	Serpinb1a
	Id3	Prf1	Cd4	Blk
	Sox4	Fasl	Icos	Il17re
	Ccr7	Cd44	Ccr7	
	Slamf6	Nkg7		
	Klf2	Il2rb		
	Sell	Cxcr3		

Supplementary Table 2: Antibodies

Compound	Fluorochrome	Clone	Company	Cat#	Dilution	Description
**PILT cells enriched population and iNKT cells sort**						
anti-CD19	Alexa Fluor 488	B4	BioLegend	115521	1:100	FACS Antibody
anti-MAIT TCR	Alexa Fluor 488	NA	NIH tetramer core facility	NA	1:400	FACS Antibody
anti-TCR γ/δ chain	Alexa Fluor 488	GL3	BioLegend	118128	1:100	FACS Antibody
anti-iNKT TCR	PE	NA	NIH tetramer core facility	NA	1:400	FACS Antibody
anti-TCR β chain	PE/Cyanine7	H57-597	BioLegend	109222	1:100	FACS Antibody
anti-CD44	eFluor 450	IM7	Invitrogen	48-0441-82	1:200	FACS Antibody
anti-PD-1	Super Bright 702	J43	Invitrogen	67-9985-82	1:100	FACS Antibody
Zombie Green Fixable Viability Kit	Zombie Green	NA	BioLegend	423112	1:400	FACS Antibody
**PILT cells staining**						
anti-CD4	Brilliant Ultra Violet 395	GK1.5	BD	115521	1:100	FACS Antibody
anti-TCR β chain	Brilliant Ultra Violet 496	H57-597	BD	749915	1:100	FACS Antibody
anti-CD8a	Brilliant Ultra Violet 737	53-6.7	BD	612759	1:400	FACS Antibody
anti-NK1.1	Brilliant Ultra Violet 805	PK136	BD	612759	1:100	FACS Antibody
anti-iNKT TCR	Brilliant Violet 421	NA	NIH tetramer core facility	NA	1:100	FACS Antibody
Zombie Aqua Fixable Viability Kit	Zombie Aqua	NA	BioLegend	423102	1:100	FACS Antibody
anti-CD19	Brilliant Violet 605	6D5	BioLegend	115540	1:100	FACS Antibody
anti-CD44	Brilliant Violet 650	IM7	BioLegend	103049	1:100	FACS Antibody
anti-CD138	Brilliant Violet 786	281-2	BD	740880	1:100	FACS Antibody
anti-TCR γ/δ chain	FITC	GL3	BioLegend	118106	1:100	FACS Antibody
anti-ROR gamma (t)	PerCP-eFluor 710	B2D	Invitrogen	46-6981-82	1:800	FACS Antibody
anti-CD24	PE	M1/69	BioLegend	101808	1:100	FACS Antibody
anti-T-bet	PE/Dazzle 594	4B10	BioLegend	644828	1:100	FACS Antibody
anti-PD-1	PE/Cyanine7	J43	Invitrogen	25-9985-82	1:100	FACS Antibody
anti-PLZF	Alexa Fluor 647	R17-809	BD	563490	1:100	FACS Antibody
anti-CD3	APC/Fire 810	17A2	BioLegend	100268	1:100	FACS Antibody
anti-PLZF	Alexa Fluor 488	Mags.21F7	BD	563490	1:100	FACS Antibody
anti-TCR γ/δ chain	Alexa Fluor 647	GL3	BioLegend	118134	1:100	FACS Antibody
Anti-F4/80	FITC	REA126	Miltenyi	130-102-327	1:100	FACS Antibody
mouse IgG1 k isotype control	Alexa Fluor 647	MOPC-31C	BD	563490	1:100	FACS Antibody
Anti-MAIT TCR	PE	NA	NIH tetramer core facility	NA	1:400	FACS Antibody
Anti-CD19	Brilliant Violet 510	1D3	BD	562956	1:100	FACS Antibody
**MS-5 cell line staining**						
anti-DLL4	APC	HMD4-1	BioLegend	130813	1:100	FACS Antibody

anti-DLL1	PE	HMD1-1	BioLegend	128307	1:100	FACS Antibody
anti-H-2Kb	APC	AF6-88.5.5.3	BioLegend	116518	1:100	FACS Antibody
anti-H-2Ld/H-2Db	PE	28-14-8	BioLegend	114507	1:100	FACS Antibody
**DN enrichment and sorting**						
anti-CD8a	Biotin	53-6.7	Invitrogen	13-0081-86	1:100	FACS Antibody
anti-CD4	Biotin	GK1.5	BioLegend	100404	1:100	FACS Antibody
anti-CD3	Biotin	17A2	BioLegend	100244	1:100	FACS Antibody
anti-TCR γ/δ chain	Biotin	GL3	BD	553176	1:100	FACS Antibody
anti-CD3e	PE	145-2C11	BD	553063	1:100	FACS Antibody
anti-CD4	PE	RM4-5	BioLegend	100512	1:100	FACS Antibody
anti-CD8a	PE	53-6.7	BioLegend	100708	1:100	FACS Antibody
anti-TCR γ/δ chain	PE	GL3	BioLegend	118108	1:100	FACS Antibody
Streptavidin, R-Phycoerythrin Conjugate	R-Phycoerythrin	NA	Invitrogen	S21388	1:100	FACS Antibody
anti-CD19	Alexa Fluor 488	B4	BioLegend	115521	1:100	FACS Antibody
Zombie Green Fixable Viability Kit	Zombie Green	NA	BioLegend	423112	1:400	FACS Antibody
anti-CD3e	APC/Cyanine7	145-2C11	BioLegend	100330	1:100	FACS Antibody
anti-CD8a	APC	QA17A07	BioLegend	100712	1:100	FACS Antibody
anti-CD4	Brilliant Violet 711	RM4-5	BioLegend	100550	1:100	FACS Antibody
anti-CD44	Brilliant Violet 650	IM7	BioLegend	103049	1:200	FACS Antibody
anti-CD25	eFluor 450	PC61.5	Invitrogen	48-0251-82	1:100	FACS Antibody
**Retrogenic ATOC**						
anti-CD4	Brilliant Ultra Violet 395	GK1.5	BD	115521	1:100	FACS Antibody
anti-TCR β chain	Brilliant Ultra Violet 496	H57-597	BD	749915	1:100	FACS Antibody
anti-CD8a	Brilliant Ultra Violet 737	53-6.7	BD	612759	1:400	FACS Antibody
anti-NK1.1	Brilliant Ultra Violet 805	PK136	BD	612759	1:100	FACS Antibody
anti-iNKT TCR	Brilliant Violet 421	NA	NIH tetramer core facility	NA	1:100	FACS Antibody
Zombie Aqua Fixable Viability Kit	Zombie Aqua	NA	BioLegend	423102	1:100	FACS Antibody
anti-CD19	Brilliant Violet 605	6D5	BioLegend	115540	1:100	FACS Antibody
anti-CD44	Brilliant Violet 650	IM7	BioLegend	103049	1:100	FACS Antibody
anti-Thy1.1	Brilliant Violet 785	OX-7	BioLegend	202553	1:100	FACS Antibody
anti-T-bet	eFluor 660	4B10	Invitrogen	50-5825-82	1:100	FACS Antibody
anti-TCR γ/δ chain	FITC	GL3	BioLegend	118106	1:100	FACS Antibody
anti-ROR gamma (t)	PerCP-eFluor 710	B2D	Invitrogen	46-6981-82	1:800	FACS Antibody
anti-PLZF	PE	R17-809	BD	564850	1:100	FACS Antibody
anti-CD3	APC/Fire 810	17A2	BioLegend	100268	1:100	FACS Antibody
**TotalSeq**						
TotalSeq-C anti-mouse Hashtag 1	NA	M1/42; 30-F11	BioLegend	155861	1:100	TotalSeq Antibody

TotalSeq-C anti-mouse Hashtag 2	NA	M1/42; 30-F11	BioLegend	155863	1:100	TotalSeq Antibody
TotalSeq-C anti-mouse Hashtag 3	NA	M1/42; 30-F11	BioLegend	155865	1:100	TotalSeq Antibody
TotalSeq-C anti-mouse Hashtag 4	NA	M1/42; 30-F11	BioLegend	155867	1:100	TotalSeq Antibody
TotalSeq-C anti-mouse Hashtag 5	NA	M1/42; 30-F11	BioLegend	155869	1:100	TotalSeq Antibody
TotalSeq-C anti-mouse Hashtag 6	NA	M1/42; 30-F11	BioLegend	155871	1:100	TotalSeq Antibody
TotalSeq-C anti-mouse TCR γ/δ chain	NA	GL3	BioLegend	118141	1:100	TotalSeq Antibody
TotalSeq-C anti-mouse CD279 (PD-1)	NA	RMP1-30	BioLegend	109127	1:100	TotalSeq Antibody
TotalSeq-C anti-mouse/human CD44	NA	IM7	BioLegend	103063	1:100	TotalSeq Antibody
TotalSeq-C anti-mouse NK-1.1	NA	PK136	BioLegend	108765	1:100	TotalSeq Antibody
TotalSeq-C0810 anti-mouse CD138 (Syndecan-1)	NA	281-2	BioLegend	352327	1:100	TotalSeq Antibody

Supplementary Table 3: List of the coding sequences of all proteins and clonotypes used in this study

Name	Coding sequences (codon optimized)
**mDLL1**	ATGGGCCGTCGGAGCGCGCTAGCCCTTGCCGTGGTCTCTGCCCTGCTGTGCCAGGTCTGGAGCTCCGGCGTATTTGAGCTGAAGCTGCAGGAGTTCGTCAATAAGAAG<br>GGGCTGCTGGGGAACCGCAACTGCTGCCGCGGGGGCTCTGGCCCGCCTTGCGCCTGCAGAACATTCTTTCGCGTATGCCTCAAGCACTACCAGGCCAGCGTGTCACCG<br>GAGCCACCCTGCACCTACGGCAGTGCTGTCACGCCAGTGCTGGGTGTCGACTCCTTCAGCCTGCCTGATGGCGCAGGCATCGACCCCGCCTTCAGTAATCCCATCCGATT<br>CCCCTTCGGCTTCACCTGGCCAGGTACCTTCTCTCTGATCATTGAAGCCCTCCATACAGACTCTCCCGATGACCTCGCAACAGAAAACCCAGAAAGACTCATCAGCCGCCT<br>GACCACACAGAGGCACCTCACTGTGGGAGAAGAATGGTCTCAGGACCTTCATAGTAGTGGACGTACAGACCTCCGGTACTCTTACCGGTTTGTGTGTGACGAGCACTAC<br>TACGGAGAAGGTTGCTCTGTGTTCTGCCGACCTCGGGATGACGCCTTTGGCCACTTCACCTGCGGGGACAGAGGTGAGAAGATGTGCGACCCTGGCTGGAAAGGCCAG<br>TACTGCACTGACCCAATCTGTCTGCCTGGGTGTGATGACCAACATGGATACTGTGACAAACCAGGAGAGTGCAAGTGCAGAGTTGGCTGGCAGGGCCGCTACTGCGAT<br>GAGTGCATCCGTTATCCAGGTTGTCTCCATGGCACATGTCAGCAACCCTGGCAGTGTAACTGCCAGGAAGGCTGGGGAGGTTTATTCTGCAACCAAGACCTGAACTACT<br>GTACTCACCATAAGCCGTGCAGGAATGGAGCCACCTGCACCAACACGGGCCAGGGGAGCTACACATGTTCCTGTCGTCCTGGGTATACAGGAGCCAATTGTGAACTGG<br>AAGTAGATGAATGTGCTCCTAGCCCCTGCAAGAACGGAGCGAGCTGCACGGACCTTGAGGACAGCTTCTCTTGCACCTGCCCTCCCGGCTTCTATGGCAAGGTCTGTGA<br>GCTGAGCGCCATGACCTGTGCAGATGGCCCTTGCTTCAATGGAGGACGATGTTCAGATAACCCTGACGGAGGCTACACTTGCCATTGCCCCTTGGGCTTCTCTGGCTTCA<br>ACTGTGAGAAGAAGATGGATCTCTGCGGCTCTTCCCCTTGTTCTAACGGTGCCAAGTGTGTGGACCTCGGCAACTCTTACCTGTGCCGGTGCCAGGCTGGCTTCTCCGG<br>GAGGTACTGCGAGGACAATGTGGATGACTGTGCCTCCTCCCCGTGTGCAAATGGGGGCACCTGCCGGGACAGTGTGAACGACTTCTCCTGTACCTGCCCACCTGGCTAC<br>ACGGGCAAGAACTGCAGCGCACCAGTCAGCAGATGTGAGCATGCACCCTGTCATAATGGTGCCACATGCCACCAGAGGGGCCAGCGCTACATGTGTGAGTGCGCCCAG<br>GGCTATGGCGGCCCCAACTGCCAGTTTCTGCTCCCTGAGCCACCACCAGGGCCCATGGTGGTGGACCTCAGTGAGAGGCATATGGAGAGCCAGGGTGGACCATTCCCT<br>TGGGTGGCAGTGTGTGCAGGAGTGGTGCTTGTCCTCCTGCTGCTGCTGGGCTGTGCTGCTGTGGTGGTCTGCGTCCGGCTGAAGCTACAGAAACACCAGCCTCCACCTG<br>AACCCTGTGGGGGAGAGACAGAAACCATGAACAACCTAGCCAATTGCCAGCGCGAGAAGGACGTTTCTGTTAGCATCATTGGGGCTACCCAGATCAAGAACACCAACA<br>AGAAGGCGGACTTTCACGGGGACCATGGAGCCGAGAAGAGCAGCTTTAAGGTCCGATACCCCACTGTGGACTATAACCTCGTTCGAGACCTCAAGGGAGATGAAGCCA<br>CGGTCAGGGATACACACAGCAAACGTGATACTAAGTGCCAGTCACAGAGCTCTGCAGGAGAAGAGAAGATCGCCCCAACACTTAGGGGTGGGGAGATTCCTGACAGA<br>AAAAGGCCAGAGTCTGTCTACTCTACTTCAAAGGACACCAAGTACCAGTCGGTGTATGTTCTGTCTGCAGAAAAGGATGAGTGTGTTATAGCGACTGAGGTGTGA
**mDLL4**	ATGACGCCTGCATCCCGTAGCGCTTGTCGCTGGGCACTACTGCTGCTGGCGGTACTGTGGCCTCAGCAGCGTGCTGCGGGCTCCGGCATCTTCCAGCTGCGGCTGCAGG<br>AGTTCGTCAACCAGCGCGGTATGCTGGCCAATGGGCAGTCCTGCGAACCGGGCTGCCGGACTTTCTTCCGCATTTGCCTTAAGCACTTCCAGGCAACCTTCTCCGAGGG<br>ACCCTGCACCTTTGGCAATGTCTCCACGCCGGTATTGGGCACCAACTCCTTCGTCGTCAGGGACAAGAATAGCGGCAGTGGTCGCAACCCTCTGCAGTTGCCCTTCAATT<br>TCACCTGGCCGGGAACCTTCTCACTCAACATCCAAGCTTGGCACACACCGGGAGACGACCTGCGGCCAGAGACTTCGCCAGGAAACTCTCTCATCAGCCAAATCATCATC<br>CAAGGCTCTCTTGCTGTGGGTAAGATTTGGCGAACAGACGAGCAAAATGACACCCTCACCAGACTGAGCTACTCTTACCGGGTCATCTGCAGTGACAACTACTATGGAG<br>AGAGCTGTTCTCGCCTATGCAAGAAGCGCGATGACCACTTCGGACATTATGAGTGTCAGCCAGATGGCAGCCTGTCCTGCCTGCCGGGCTGGACTGGGAAGTACTGTG<br>ACCAGCCTATATGTCTTTCTGGCTGTCATGAGCAGAATGGTTACTGCAGCAAGCCAGATGAGTGCATCTGCCGTCCAGGTTGGCAGGGTCGCCTGTGCAATGAATGTAT<br>CCCCCACAATGGCTGTCGTCATGGCACCTGCAGCATCCCCTGGCAGTGTGCCTGCGATGAGGGATGGGGAGGTCTGTTTTGTGACCAAGATCTCAACTACTGTACTCAC<br>CACTCTCCGTGCAAGAATGGATCAACGTGTTCCAACAGTGGGCCAAAGGGTTATACCTGCACCTGTCTCCCAGGCTACACTGGTGAGCACTGTGAGCTGGGACTCAGCA<br>AGTGTGCCAGCAACCCCTGTCGAAATGGTGGCAGCTGTAAGGACCAGGAGAATAGCTACCACTGCCTGTGTCCCCCAGGCTACTATGGCCAGCACTGTGAGCATAGTAC<br>CTTGACCTGTGCGGACTCACCATGTTTTAATGGCGGCTCTTGCCGGGAGCGCAACCAGGGGTCCAGTTATGCCTGCGAATGCCCCCCCAACTTTACCGGCTCTAACTGTG<br>AGAAGAAAGTAGACAGGTGTACCAGCAACCCGTGTGCCAATGGAGGCCAGTGCCAGAACAGAGGTCCAAGCCGAACCTGCCGCTGCCGGCCTGGATTCACAGGCACC<br>CACTGTGAACTGCACATCAGCGATTGTGCCCGAAGTCCCTGTGCCCACGGGGGCACTTGCCACGATCTGGAGAATGGGCCTGTGTGCACCTGCCCCGCTGGCTTCTCTG<br>GCAGGCGCTGCGAGGTGCGGATAACCCACGATGCCTGTGCCTCCGGACCCTGCTTCAATGGGGCCACCTGCTACACTGGCCTCTCCCCAAACAACTTCGTCTGCAACTGT<br>CCTTATGGCTTTGTGGGCAGCCGCTGCGAGTTTCCCGTGGGCTTGCCACCCAGCTTCCCCTGGGTAGCTGTCTCGCTGGGCGTGGGGCTAGTGGTACTGCTGGTGCTGC<br>TGGTCATGGTGGTAGTGGCTGTGCGGCAGCTGCGGCTTCGGAGGCCCGATGACGAGAGCAGGGAAGCCATGAACAATCTGTCAGACTTCCAGAAGGACAACCTAATC<br>CCTGCCGCCCAGCTCAAAAACACAAACCAGAAGAAGGAGCTGGAAGTGGACTGTGGTCTGGACAAGTCCAATTGTGGCAAACTGCAGAACCACACATTGGACTACAAT<br>CTAGCCCCGGGACTCCTAGGACGGGGCGGCATGCCTGGGAAGTATCCTCACAGTGACAAGAGCTTAGGAGAGAAGGTGCCACTTCGGTTACACAGTGAGAAGCCAGA<br>GTGTCGAATATCAGCCATTTGCTCTCCCAGGGACTCTATGTACCAATCAGTGTGTTTGATATCAGAAGAGAGGAACGAGTGTGTGATTGCCACAGAGGTATGA
**mH2-Kb**	ATGGTACCGTGCACGCTGCTCCTGCTGTTGGCGGCCGCCCTGGCTCCGACTCAGACCCGCGCGGGCCCACACTCGCTGAGGTATTTCGTCACCGCCGTGTCCCGGCCCG<br>GCCTCGGGGAGCCCCGGTACATGGAAGTCGGCTACGTGGACGACACGGAGTTCGTGCGCTTCGACAGCGACGCGGAGAATCCGAGATATGAGCCGCGGGCGCGGTG<br>GATGGAGCAGGAGGGGCCCGAGTATTGGGAGCGGGAGACACAGAAAGCCAAGGGCAATGAGCAGAGTTTCCGAGTGGACCTGAGGACCCTGCTCGGCTACTACAAC<br>CAGAGCAAGGGCGGCTCTCACACTATTCAGGTGATCTCTGGCTGTGAAGTGGGGTCCGACGGGCGACTCCTCCGCGGGTACCAGCAGTACGCCTACGACGGCTGCGAT<br>TACATCGCCCTGAACGAAGACCTGAAAACGTGGACGGCGGCGGACATGGCGGCGCTGATCACCAAACACAAGTGGGAGCAGGCTGGTGAAGCAGAGAGACTCAGGG<br>CCTACCTGGAGGGCACGTGCGTGGAGTGGCTCCGCAGATACCTGAAGAACGGGAACGCGACGCTGCTGCGCACAGATTCCCCAAAGGCCCATGTGACCCATCACAGCA<br>GACCTGAAGATAAAGTCACCCTGAGGTGCTGGGCCCTGGGCTTCTACCCTGCTGACATCACCCTGACCTGGCAGTTGAATGGGGAGGAGCTGATCCAGGACATGGAGC<br>TTGTGGAGACCAGGCCTGCAGGGGATGGAACCTTCCAGAAGTGGGCATCTGTGGTGGTGCCTCTTGGGAAGGAGCAGTATTACACATGCCATGTGTACCATCAGGGGC<br>TGCCTGAGCCCCTCACCCTGAGATGGGAGCCTCCTCCATCCACTGTCTCCAACATGGCGACCGTTGCTGTTCTGGTTGTCCTTGGAGCTGCAATAGTCACTGGAGCTGTG<br>GTGGCTTTTGTGATGAAGATGAGAAGGAGAAACACAGGTGGAAAAGGAGGGGACTATGCTCTGGCTCCAGGCTCCCAGACCTCTGATCTGTCTCTCCCAGATTGTAAA<br>GTGATGGTTCATGACCCTCATTCTCTAGCGTGA
**mH2-Db**	ATGGGGGCGATGGCTCCGCGCACGCTGCTCCTGCTGCTGGCGGCCGCCCTGGCCCCGACTCAGACCCGCGCGGGCCCACACTCGATGCGGTATTTCGAGACCGCCGTG<br>TCCCGGCCCGGCCTCGAGGAGCCCCGGTACATCTCTGTCGGCTATGTGGACAACAAGGAGTTCGTGCGCTTCGACAGCGACGCGGAGAATCCGAGATATGAGCCGCGG<br>GCGCCGTGGATGGAGCAGGAGGGGCCGGAGTATTGGGAGCGGGAAACACAGAAAGCCAAGGGCCAAGAGCAGTGGTTCCGAGTGAGCCTGAGGAACCTGCTCGGC<br>TACTACAACCAGAGCGCGGGCGGCTCTCACACACTCCAGCAGATGTCTGGCTGTGACTTGGGGTCGGACTGGCGCCTCCTCCGCGGGTACCTGCAGTTCGCCTATGAAG<br>GCCGCGATTACATCGCCCTGAACGAAGACCTGAAAACGTGGACGGCGGCGGACATGGCGGCGCAGATCACCCGACGCAAGTGGGAGCAGAGTGGTGCTGCAGAGCA<br>TTACAAGGCCTACCTGGAGGGCGAGTGCGTGGAGTGGCTCCACAGATACCTGAAGAACGGGAACGCGACGCTGCTGCGCACAGATTCCCCAAAGGCACATGTGACCC<br>ATCACCCCAGATCTAAAGGTGAAGTCACCCTGAGGTGCTGGGCCCTGGGCTTCTACCCTGCTGACATCACCCTGACCTGGCAGTTGAATGGGGAGGAGCTGACCCAGG<br>ACATGGAGCTTGTGGAGACCAGGCCTGCAGGGGATGGAACCTTCCAGAAGTGGGCATCTGTGGTGGTGCCTCTTGGGAAGGAGCAGAATTACACATGCCGTGTGTAC<br>CATGAGGGGCTGCCTGAGCCCCTCACCCTGAGATGGGAGCCTCCTCCGTCCACTGACTCTTACATGGTGATCGTTGCTGTTCTGGGTGTCCTTGGAGCTATGGCCATCAT<br>TGGAGCTGTGGTGGCTTTTGTGATGAAGAGAAGGAGAAACACAGGTGGAAAAGGAGGGGACTATGCTCTGGCTCCAGGCTCCCAGAGCTCTGAAATGTCTCTCCGAG<br>ATTGTAAAGCGTGA
**Clonotype iNKT TCRα**	ATGAAAAAGCGCCTGAGTGCCTGCTGGGTGGTCTTGTGGCTGCATTATCAGTGGGTGGCTGGCAAGACCCAAGTGGAGCAGAGTCCTCAGTCCCTGGTTGTCCGTCAG<br>GGAGAGAACTGCGTCCTTCAATGTAATTACAGTGTGACCCCCGACAACCACTTAAGGTGGTTCAAACAGGACACAGGCAAAGGTCTTGTGTCCCTGACAGTCCTGGTTG<br>ACCAAAAAGACAAAACGTCAAATGGGAGATACTCAGCAACTCTGGATAAAGATGCAAAGCACAGCACGCTGCACATCACAGCCACCCTGCTGGATGACACTGCCACCT<br>ACATCTGTGTGGTGGGCGATAGAGGTTCAGCCTTAGGGAGGCTGCATTTTGGAGCTGGGACTCAGCTGATTGTCATACCTGACATCCAGAATCCGGAACCTGCTGTGTA<br>CCAGTTAAAGGATCCTCGGTCTCAGGACAGCACCCTCTGCCTGTTCACCGACTTTGACTCCCAAATCAATGTGCCGAAAACCATGGAATCTGGAACGTTCATCACGGACA<br>AAACTGTGCTGGACATGAAAGCTATGGATTCCAAGAGCAATGGGGCCATTGCCTGGAGCAACCAGACAAGCTTCACCTGCCAAGATATCTTCAAAGAGACCAACGCCA<br>CCTACCCCAGTTCAGACGTTCCCTGTGATGCCACGTTGACCGAGAAAAGCTTTGAAACAGATATGAACCTAAACTTTCAAAACCTGTCAGTTATGGGACTCCGAATCCTC<br>CTGCTGAAAGTAGCGGGATTTAACCTGCTCATGACGCTGAGGCTGTGGTCCTGA

**Clonotype iNKT TCRβ**	ATGAGAGTTAGGCTCATCTCTGCTGTGGTGCTGTGTTTCCTAGGAACAGGCCTTGTGGACATGAAAGTAACCCAGATGCCAAGATACCTGATCAAAAGAATGGGAGAG<br>AATGTTTTGCTGGAATGTGGACAGGACATGAGCCATGAAACAATGTACTGGTATCGACAAGACCCTGGTCTGGGGCTACAGCTGATTTATATCTCATACGATGTTGATA<br>GTAACAGCGAAGGAGACATCCCTAAAGGATACAGGGTCTCACGGAAGAAGCGGGAGCATTTCTCCCTGATTCTGGATTCTGCTAAAACAAACCAGACATCTGTGTACTT<br>CTGTGCTAGCAGTTTGGGAAATACGCTCTATTTTGGAGAAGGAAGCCGGCTCATTGTTGTAGAGGATCTGAGAAATGTGACTCCACCCAAGGTCTCCTTGTTTGAGCCAT<br>CAAAAGCAGAGATTGCAAACAAACAAAAGGCTACCCTCGTGTGCTTGGCCAGGGGCTTCTTCCCTGACCACGTGGAGCTGAGCTGGTGGGTGAATGGCAAGGAGGTCC<br>ACAGTGGGGTCAGCACGGACCCTCAGGCCTACAAGGAGAGCAATTATAGCTACTGCCTGAGCAGCCGCCTGAGGGTCTCTGCTACCTTCTGGCACAATCCTCGCAACCA<br>CTTCCGCTGCCAAGTGCAGTTCCATGGGCTTTCAGAGGAGGACAAGTGGCCAGAGGGCTCACCCAAACCTGTCACACAGAACATCAGTGCAGAGGCCTGGGGCCGAGC<br>AGACTGTGGGATTACCTCAGCATCCTATCAACAAGGGGTCTTGTCTGCCACCATCCTCTATGAGATCCTGCTAGGGAAAGCCACCCTGTATGCTGTGCTTGTCAGTACAC<br>TGGTGGTGATGGCTATGGTCAAAAGAAAGAACTCATGA
**Clonotype OTI TCRα**	ATGGACAAGATCCTGACAGCATCGTTTTTACTCCTAGGCCTTCACCTAGCTGGGGTGAATGGCCAGCAGCAGGAGAAACGTGACCAGCAGCAGGTGAGACAAAGTCCC<br>CAATCTCTGACAGTCTGGGAAGGAGAGACCGCAATTCTGAACTGCAGTTATGAGGACAGCACTTTTAACTACTTCCCATGGTACCAGCAGTTCCCTGGGGAAGGCCCTG<br>CACTCCTGATATCCATACGTTCAGTGTCCGATAAAAAGGAAGATGGACGATTCACAATCTTCTTCAATAAAAGGGAGAAAAAGCTCTCCTTGCACATCACAGACTCTCAG<br>CCTGGAGACTCAGCTACCTACTTCTGTGCAGCAAGTGACAACTATCAGTTGATCTGGGGCTCTGGGACCAAGCTAATTATAAAGCCAGACATCCAGAATCCGGAACCTG<br>CTGTGTACCAGTTAAAGGATCCTCGGTCTCAGGACAGCACCCTCTGCCTGTTCACCGACTTTGACTCCCAAATCAATGTGCCGAAAACCATGGAATCTGGAACGTTCATC<br>ACGGACAAAACTGTGCTGGACATGAAAGCTATGGATTCCAAGAGCAATGGGGCCATTGCCTGGAGCAACCAGACAAGCTTCACCTGCCAAGATATCTTCAAAGAGACC<br>AACGCCACCTACCCCAGTTCAGACGTTCCCTGTGATGCCACGTTGACCGAGAAAAGCTTTGAAACAGATATGAACCTAAACTTTCAAAACCTGTCAGTTATGGGACTCCG<br>AATCCTCCTGCTGAAAGTAGCGGGATTTAACCTGCTCATGACGCTGAGGCTGTGGTCCTGA
**Clonotype OTI TCRβ**	ATGTCTAACACTGTCCTCGCTGATTCTGCCTGGGGCATCACCCTGCTATCTTGGGTTACTGTCTTTCTCTTGGGAACAAGTTCAGCAGATTCTGGGGTTGTCCAGTCTCCA<br>AGACACATAATCAAAGAAAAGGGAGGAAGGTCCGTTCTGACGTGTATTCCCATCTCTGGACATAGCAATGTGGTCTGGTACCAGCAGACTCTGGGGAAGGAATTAAAG<br>TTCCTTATTCAGCATTATGAAAAGGTGGAGAGAGACAAAGGATTCCTACCCAGCAGATTCTCAGTCCAACAGTTTGATGACTATCACTCTGAAATGAACATGAGTGCCTT<br>GGAACTGGAGGACTCTGCTATGTACTTCTGTGCCAGCTCTCGGGCCAATTATGAACAGTACTTCGGTCCCGGCACCAGGCTCACGGTTTTAGAGGATCTGAGAAATGTG<br>ACTCCACCCAAGGTCTCCTTGTTTGAGCCATCAAAAGCAGAGATTGCAAACAAACAAAAGGCTACCCTCGTGTGCTTGGCCAGGGGCTTCTTCCCTGACCACGTGGAGCT<br>GAGCTGGTGGGTGAATGGCAAGGAGGTCCACAGTGGGGTCAGCACGGACCCTCAGGCCTACAAGGAGAGCAATTATAGCTACTGCCTGAGCAGCCGCCTGAGGGTCT<br>CTGCTACCTTCTGGCACAATCCTCGAAACCACTTCCGCTGCCAAGTGCAGTTCCATGGGCTTTCAGAGGAGGACAAGTGGCCAGAGGGCTCACCCAAACCTGTCACACA<br>GAACATCAGTGCAGAGGCCTGGGGCCGAGCAGACTGTGGAATCACTTCAGCATCCTATCATCAGGGGGTTCTGTCTGCAACCATCCTCTATGAGATCCTACTGGGGAAG<br>GCCACCCTATATGCTGTGCTGGTCAGTGGCCTGGTGCTGATGGCTATGGTCAAGAAAAAAAATTCCTGA
**Clonotype 1 TCRα**	ATGCGTCCTGTCACCTGCTCAGTTCTTGTGCTCCTCCTAATGCTCAGGAGGAGCAATGGCGATGGAGACTCCGTGACCCAGACAGAAGGCCTGGTCACTCTCACAGAAG<br>GGTTGCCTGTGATGCTGAACTGCACCTATCAGACTATTTACTCAAATCCTTTCCTTTTCTGGTATGTGCAACATCTCAATGAATCCCCTCGGCTACTCCTGAAGAGCTTCAC<br>AGACAACAAGAGGACCGAGCACCAAGGGTTCCACGCCACTCTCCATAAGAGCAGCAGCTCCTTCCATCTGCAGAAGTCCTCAGCGCAGCTGTCAGACTCTGCCCTGTAC<br>TACTGTGCTCTGAGCGGGAATTACAACGTGCTTTACTTCGGATCTGGCACCAAACTCACTGTAGAGCCAAACATCCAGAATCCGGAACCTGCTGTGTACCAGTTAAAGGA<br>TCCTCGGTCTCAGGACAGCACCCTCTGCCTGTTCACCGACTTTGACTCCCAAATCAATGTGCCGAAAACCATGGAATCTGGAACGTTCATCACGGACAAAACTGTGCTGG<br>ACATGAAAGCTATGGATTCCAAGAGCAATGGGGCCATTGCCTGGAGCAACCAGACAAGCTTCACCTGCCAAGATATCTTCAAAGAGACCAACGCCACCTACCCCAGTTC<br>AGACGTTCCCTGTGATGCCACGTTGACCGAGAAAAGCTTTGAAACAGATATGAACCTAAACTTTCAAAACCTGTCAGTTATGGGACTCCGAATCCTCCTGCTGAAAGTAG<br>CGGGATTTAACCTGCTCATGACGCTGAGGCTGTGGTCCTGA
**Clonotype 1 TCRβ**	ATGGCTACAAGGCTCCTCTGTTACACAGTACTTTGTCTCCTGGGTGCAAGAATTTTGAACTCAAAAGTCATTCAGACTCCAAGATATCTGGTGAAAGGGCAAGGACAAAA<br>AGCAAAGATGAGGTGTATCCCTGAAAAGGGACATCCAGTTGTATTCTGGTATCAACAAAATAAGAACAATGAGTTTAAATTTTTGATTAACTTTCAGAATCAAGAAGTTC<br>TTCAGCAAATAGACATGACTGAAAAACGATTCTCTGCTGAGTGTCCTTCAAACTCACCTTGCAGCCTAGAAATTCAGTCCTCTGAGGCAGGAGACTCAGCACTGTACCTC<br>TGTGCCAGCAGGGACTGGGGGGGGAACTATGCTGAGCAGTTCTTCGGACCAGGGACACGACTCACCGTCCTAGAGGATCTGAGAAATGTGACTCCACCCAAGGTCTCC<br>TTGTTTGAGCCATCAAAAGCAGAGATTGCAAACAAACAAAAGGCTACCCTCGTGTGCTTGGCCAGGGGCTTCTTCCCTGACCACGTGGAGCTGAGCTGGTGGGTGAAT<br>GGCAAGGAGGTCCACAGTGGGGTCAGCACGGACCCTCAGGCCTACAAGGAGAGCAATTATAGCTACTGCCTGAGCAGCCGCCTGAGGGTCTCTGCTACCTTCTGGCAC<br>AATCCTCGAAACCACTTCCGCTGCCAAGTGCAGTTCCATGGGCTTTCAGAGGAGGACAAGTGGCCAGAGGGCTCACCCAAACCTGTCACACAGAACATCAGTGCAGAG<br>GCCTGGGGCCGAGCAGACTGTGGAATCACTTCAGCATCCTATCATCAGGGGGTTCTGTCTGCAACCATCCTCTATGAGATCCTACTGGGGAAGGCCACCCTATATGCTGT<br>GCTGGTCAGTGGCCTGGTGCTGATGGCTATGGTCAAGAAAAAAAATTCCTGA
**Clonotype 2 TCRα**	ATGCTGATTCTAAGCCTGCTGGGAGCAGCCTTTGGCTCCATTTGTTTTGCAACCAGCATGGCCCAGAAGGTAACACAGACTCAGACTTCAATTTCTGTGATGGAGAAGAC<br>AACGGTGACAATGGACTGTGTGTATGAAACCCGGGACAGTTCTTACTTCTTATTCTGGTACAAGCAAACAGCAAGTGGGGAAATAGTTTTCCTTATTCGTCAGGACTCTT<br>ACAAAAAGGAAAATGCAACAGAAGGTCATTATTCTCTGAACTTTCAGAAGCCAAAAAGTTCCATCGGACTCATCATCACTGCCACACAGATTGAGGACTCAGCAGTATA<br>TTTCTGTGCTATGAGAGGGACTGGAGGCAATAATAAGCTGACTTTTGGTCAAGGAACCGTTCTGAGTGTTATACCAGACATCCAGAATCCGGAACCTGCTGTGTACCAG<br>TTAAAGGATCCTCGGTCTCAGGACAGCACCCTCTGCCTGTTCACCGACTTTGACTCCCAAATCAATGTGCCGAAAACCATGGAATCTGGAACGTTCATCACGGACAAAAC<br>TGTGCTGGACATGAAAGCTATGGATTCCAAGAGCAATGGGGCCATTGCCTGGAGCAACCAGACAAGCTTCACCTGCCAAGATATCTTCAAAGAGACCAACGCCACCTAC<br>CCCAGTTCAGACGTTCCCTGTGATGCCACGTTGACCGAGAAAAGCTTTGAAACAGATATGAACCTAAACTTTCAAAACCTGTCAGTTATGGGACTCCGAATCCTCCTGCT<br>GAAAGTAGCGGGATTTAACCTGCTCATGACGCTGAGGCTGTGGTCCTGA
**Clonotype 2 TCRβ**	ATGAACAAGTGGGTTTTCTGCTGGGTAACCCTTTGTCTCCTTACTGTAGAGACCACACATGGTGATGGTGGCATCATTACTCAGACACCCAAATTCCTGATTGGTCAGGA<br>AGGGCAAAAACTGACCTTGAAATGTCAACAGAATTTCAATCATGATACAATGTACTGGTACCGACAGGATTCAGGGAAAGGATTGAGACTGATCTACTATTCAATAACT<br>GAAAACGATCTTCAAAAAGGCGATCTATCTGAAGGCTATGATGCGTCTCGAGAGAAGAAGTCATCTTTTTCTCTCACTGTGACATCTGCCCAGAAGAACGAGATGGCCG<br>TTTTTCTCTGTGCCAGCAGTCCGGGACTGGGGGGGCTGGAACAGTACTTCGGTCCCGGCACCAGGCTCACGGTTTTAGAGGATCTGAGAAATGTGACTCCACCCAAGGT<br>CTCCTTGTTTGAGCCATCAAAAGCAGAGATTGCAAACAAACAAAAGGCTACCCTCGTGTGCTTGGCCAGGGGCTTCTTCCCTGACCACGTGGAGCTGAGCTGGTGGGTG<br>AATGGCAAGGAGGTCCACAGTGGGGTCAGCACGGACCCTCAGGCCTACAAGGAGAGCAATTATAGCTACTGCCTGAGCAGCCGCCTGAGGGTCTCTGCTACCTTCTGG<br>CACAATCCTCGAAACCACTTCCGCTGCCAAGTGCAGTTCCATGGGCTTTCAGAGGAGGACAAGTGGCCAGAGGGCTCACCCAAACCTGTCACACAGAACATCAGTGCAG<br>AGGCCTGGGGCCGAGCAGACTGTGGAATCACTTCAGCATCCTATCATCAGGGGGTTCTGTCTGCAACCATCCTCTATGAGATCCTACTGGGGAAGGCCACCCTATATGC<br>TGTGCTGGTCAGTGGCCTGGTGCTGATGGCTATGGTCAAGAAAAAAAATTCCTGA
**Clonotype 3 TCRα**	ATGGACAAGATCCTGACAGCATTGTTTTTACTTCTAGGTCTTCACCTAGCTGGGGTGAGTGGCCAGCAGGAGAAACATGACCAGCAGCAGGTGAGACAAAGTTCCCAAT<br>CTCTGACAGTCTGGGAAGGAGAGACCGCAATTCTGAACTGCAGTTATGAGAACAGTGCTTTTGACTACTTCCCATGGTACCAGCAGTTCCCTGGGGAAGGCCCTGCTCT<br>CCTGATAGCCATACGTTCAGTGTCCGATAAAAAGGAAGATGGACGATTCACAATCTTCTTCAATAAAAGGGAGAAAAATCTCTCCTTGCACATCAAAGACTCTCAGCCTG<br>GAGACTCAGCCACCTACTTCTGTGCAGCAAGTCCTAATGCAGGTGCCAAGCTCACATTCGGAGGGGGAACAAGGTTAACGGTCAGACCCGACATCCAGAATCCGGAAC<br>CTGCTGTGTACCAGTTAAAGGATCCTCGGTCTCAGGACAGCACCCTCTGCCTGTTCACCGACTTTGACTCCCAAATCAATGTGCCGAAAACCATGGAATCTGGAACGTTC<br>ATCACGGACAAAACTGTGCTGGACATGAAAGCTATGGATTCCAAGAGCAATGGGGCCATTGCCTGGAGCAACCAGACAAGCTTCACCTGCCAAGATATCTTCAAAGAG<br>ACCAACGCCACCTACCCCAGTTCAGACGTTCCCTGTGATGCCACGTTGACCGAGAAAAGCTTTGAAACAGATATGAACCTAAACTTTCAAAACCTGTCAGTTATGGGACT<br>CCGAATCCTCCTGCTGAAAGTAGCGGGATTTAACCTGCTCATGACGCTGAGGCTGTGGTCCTGA
**Clonotype 3 TCRβ**	ATGTCTAACACTGTCCTCGCTGATTCTGCCTGGGGCATCACCCTGCTATCTTGGGTTACTGTCTTTCTCTTGGGAACAAGTTCAGCAGATTCTGGGGTTGTCCAGTCTCCA<br>AGACACATAATCAAAGAAAAGGGAGGAAGGTCCGTTCTGACGTGTATTCCCATCTCTGGACATAGCAATGTGGTCTGGTACCAGCAGACTCTGGGGAAGGAATTAAAG<br>TTCCTTATTCAGCATTATGAAAAGGTGGAGAGAGACAAAGGATTCCTACCCAGCAGATTCTCAGTCCAACAGTTTGATGACTATCACTCTGAAATGAACATGAGTGCCTT<br>GGAACTGGAGGACTCTGCTATGTACTTCTGTGCCAGCTCCCAGGACAGGGGGCGCTCCTATGAACAGTACTTCGGTCCCGGCACCAGGCTCACGGTTTTAGAGGATCTG<br>AGAAATGTGACTCCACCCAAGGTCTCCTTGTTTGAGCCATCAAAAGCAGAGATTGCAAACAAACAAAAGGCTACCCTCGTGTGCTTGGCCAGGGGCTTCTTCCCTGACCA<br>CGTGGAGCTGAGCTGGTGGGTGAATGGCAAGGAGGTCCACAGTGGGGTCAGCACGGACCCTCAGGCCTACAAGGAGAGCAATTATAGCTACTGCCTGAGCAGCCGCC<br>TGAGGGTCTCTGCTACCTTCTGGCACAATCCTCGAAACCACTTCCGCTGCCAAGTGCAGTTCCATGGGCTTTCAGAGGAGGACAAGTGGCCAGAGGGCTCACCCAAACC<br>TGTCACACAGAACATCAGTGCAGAGGCCTGGGGCCGAGCAGACTGTGGAATCACTTCAGCATCCTATCATCAGGGGGTTCTGTCTGCAACCATCCTCTATGAGATCCTA<br>CTGGGGAAGGCCACCCTATATGCTGTGCTGGTCAGTGGCCTGGTGCTGATGGCTATGGTCAAGAAAAAAAATTCCTGA

**Clonotype 4 TCRα**	ATGAAATCCTTGAGTGTTTCACTAGTGGTCCTGTGGCTCCAGTTAAACTGCGTGAGGAGCCAGCAGAAGGTGCAGCAGAGCCCAGAATCCCTCAGTGTCCCAGAGGGA<br>GGCATGGCCTCTCTCAACTGCACTTCAAGTGATCGTAATTTTCAGTACTTCTGGTGGTACAGACAGCATTCTGGAGAAGGCCCCAAGGCACTGATGTCAATCTTCTCTGA<br>TGGTGACAAGAAAGAAGGCAGATTCACAGCTCACCTCAATAAGGCCAGCCTGCATGTTTCCCTGCACATCAGAGACTCCCAGCCCAGTGACTCCGCTCTCTACTTCTGTG<br>CAGCTTTGTCTAATTACAACGTGCTTTACTTCGGATCTGGCACCAAACTCACTGTAGAGCCAAACATCCAGAATCCGGAACCTGCTGTGTACCAGTTAAAGGATCCTCGG<br>TCTCAGGACAGCACCCTCTGCCTGTTCACCGACTTTGACTCCCAAATCAATGTGCCGAAAACCATGGAATCTGGAACGTTCATCACGGACAAAACTGTGCTGGACATGAA<br>AGCTATGGATTCCAAGAGCAATGGGGCCATTGCCTGGAGCAACCAGACAAGCTTCACCTGCCAAGATATCTTCAAAGAGACCAACGCCACCTACCCCAGTTCAGACGTT<br>CCCTGTGATGCCACGTTGACCGAGAAAAGCTTTGAAACAGATATGAACCTAAACTTTCAAAACCTGTCAGTTATGGGACTCCGAATCCTCCTGCTGAAAGTAGCGGGATT<br>TAACCTGCTCATGACGCTGAGGCTGTGGTCCTGA
**Clonotype 4 TCRβ**	ATGGGCTCCAGGCTCTTTCTGGTCTTGAGCCTCCTGTGTACAAAACACATGGAGGCTGCAGTCACCCAAAGCCCTAGAAACAAGGTGACAGTAACAGGAGGAAACGTG<br>ACATTGAGCTGTCGCCAGACTAATAGCCACAACTACATGTACTGGTATCGGCAGGACACTGGGCATGGGCTGAGGCTGATCCATTACTCATATGGTGCTGGCAACCTTC<br>AAATAGGAGATGTCCCTGATGGGTACAAGGCCACCAGAACAACGCAAGAAGACTTCTTCCTCCTGCTGGAATTGGCTTCTCCCTCTCAGACATCTTTGTACTTCTGTGCC<br>AGCAGTGATTCAGGGGGCGGCACACAGTACTTTGGGCCAGGCACTCGGCTCCTCGTGTTAGAGGATCTGAGAAATGTGACTCCACCCAAGGTCTCCTTGTTTGAGCCAT<br>CAAAAGCAGAGATTGCAAACAAACAAAAGGCTACCCTCGTGTGCTTGGCCAGGGGCTTCTTCCCTGACCACGTGGAGCTGAGCTGGTGGGTGAATGGCAAGGAGGTCC<br>ACAGTGGGGTCAGCACGGACCCTCAGGCCTACAAGGAGAGCAATTATAGCTACTGCCTGAGCAGCCGCCTGAGGGTCTCTGCTACCTTCTGGCACAATCCTCGAAACCA<br>CTTCCGCTGCCAAGTGCAGTTCCATGGGCTTTCAGAGGAGGACAAGTGGCCAGAGGGCTCACCCAAACCTGTCACACAGAACATCAGTGCAGAGGCCTGGGGCCGAGC<br>AGACTGTGGAATCACTTCAGCATCCTATCATCAGGGGGTTCTGTCTGCAACCATCCTCTATGAGATCCTACTGGGGAAGGCCACCCTATATGCTGTGCTGGTCAGTGGCC<br>TGGTGCTGATGGCTATGGTCAAGAAAAAAAATTCCTGA
**Clonotype 5 TCRα**	ATGCTCCTGGCGCTCCTCCCAGTGCTGGGGATACACTTTGTCCTGAGAGATGCCCAAGCTCAGTCAGTGACACAGCCCGATGCTCGCGTCACTGTCTCTGAAGGAGCCTC<br>TCTGCAGCTGAGATGCAAGTATTCCTACTCTGCGACACCTTATCTGTTCTGGTATGTCCAGTACCCGCGGCAGGGGCTGCAGCTGCTCCTCAAGTACTATTCAGGAGACC<br>CAGTGGTTCAAGGAGTGAACAGCTTCGAGGCTGAGTTCAGCAAGAGTAACTCTTCCTTCCACCTGCAGAAAGCCTCTGTGCACTGGAGCGACTCGGCTGTGTACTTCTG<br>TGCTCTAGACACCAATACAGGCAAATTAACCTTTGGGGATGGGACCGTGCTCACAGTGAAGCCAAACATCCAGAATCCGGAACCTGCTGTGTACCAGTTAAAGGATCCT<br>CGGTCTCAGGACAGCACCCTCTGCCTGTTCACCGACTTTGACTCCCAAATCAATGTGCCGAAAACCATGGAATCTGGAACGTTCATCACGGACAAAACTGTGCTGGACAT<br>GAAAGCTATGGATTCCAAGAGCAATGGGGCCATTGCCTGGAGCAACCAGACAAGCTTCACCTGCCAAGATATCTTCAAAGAGACCAACGCCACCTACCCCAGTTCAGAC<br>GTTCCCTGTGATGCCACGTTGACCGAGAAAAGCTTTGAAACAGATATGAACCTAAACTTTCAAAACCTGTCAGTTATGGGACTCCGAATCCTCCTGCTGAAAGTAGCGGG<br>ATTTAACCTGCTCATGACGCTGAGGCTGTGGTCCTGA
**Clonotype 5 TCRβ**	ATGAGAGTTAGGCTCATCTCTGCTGTGGTGCTGTGTTTCCTAGGAACAGGCCTTGTGGACATGAAAGTAACCCAGATGCCAAGATACCTGATCAAAAGAATGGGAGAG<br>AATGTTTTGCTGGAATGTGGACAGGACATGAGCCATGAAACAATGTACTGGTATCGACAAGACCCTGGTCTGGGGCTACAGCTGATTTATATCTCATACGATGTTGATA<br>GTAACAGCGAAGGAGACATCCCTAAAGGATACAGGGTCTCACGGAAGAAGCGGGAGCATTTCTCCCTGATTCTGGATTCTGCTAAAACAAACCAGACATCTGTGTACTT<br>CTGTGCTAGCAGTTTATCACAGGGGACCGGGCAGCTCTACTTTGGTGAAGGCTCAAAGCTGACAGTGCTGGAGGATCTGAGAAATGTGACTCCACCCAAGGTCTCCTTG<br>TTTGAGCCATCAAAAGCAGAGATTGCAAACAAACAAAAGGCTACCCTCGTGTGCTTGGCCAGGGGCTTCTTCCCTGACCACGTGGAGCTGAGCTGGTGGGTGAATGGC<br>AAGGAGGTCCACAGTGGGGTCAGCACGGACCCTCAGGCCTACAAGGAGAGCAATTATAGCTACTGCCTGAGCAGCCGCCTGAGGGTCTCTGCTACCTTCTGGCACAAT<br>CCTCGAAACCACTTCCGCTGCCAAGTGCAGTTCCATGGGCTTTCAGAGGAGGACAAGTGGCCAGAGGGCTCACCCAAACCTGTCACACAGAACATCAGTGCAGAGGCC<br>TGGGGCCGAGCAGACTGTGGAATCACTTCAGCATCCTATCATCAGGGGGTTCTGTCTGCAACCATCCTCTATGAGATCCTACTGGGGAAGGCCACCCTATATGCTGTGCT<br>GGTCAGTGGCCTGGTGCTGATGGCTATGGTCAAGAAAAAAAATTCCTGA
**Clonotype 6 TCRα**	ATGGAGAGGAACCTGGGAGCTGTGCTGGGGATTCTGTGGGTGCAGATTTGCTGGGTGAGCGGAGATAAGGTGAAGCAAAGTCCCTCAGCGCTGAGTCTCCAAGAGG<br>GAACCAATTCTGCTCTGAGATGCAATTTTTCTATCGCTGCGACAACTGTGCAGTGGTTCCTACAGAATCCCAGGGGCAGCCTCATGAATCTTTTTTACCTGGTGCCAGGA<br>ACAAAGGAGAATGGGAGGTTAAAGTCAGCATTTGATTCTAAGGAGAGCTACAGCACCCTGCACATTAGGGATGCCCAGCTGGAGGACTCAGGCACTTACTTCTGTGCT<br>GCTGAGGATGGGAGCAGTGGCAACAAGCTCATCTTTGGAATTGGGACTCTGCTTTCTGTCAAGCCAAACATCCAGAATCCGGAACCTGCTGTGTACCAGTTAAAGGATC<br>CTCGGTCTCAGGACAGCACCCTCTGCCTGTTCACCGACTTTGACTCCCAAATCAATGTGCCGAAAACCATGGAATCTGGAACGTTCATCACGGACAAAACTGTGCTGGAC<br>ATGAAAGCTATGGATTCCAAGAGCAATGGGGCCATTGCCTGGAGCAACCAGACAAGCTTCACCTGCCAAGATATCTTCAAAGAGACCAACGCCACCTACCCCAGTTCAG<br>ACGTTCCCTGTGATGCCACGTTGACCGAGAAAAGCTTTGAAACAGATATGAACCTAAACTTTCAAAACCTGTCAGTTATGGGACTCCGAATCCTCCTGCTGAAAGTAGCG<br>GGATTTAACCTGCTCATGACGCTGAGGCTGTGGTCCTGA
**Clonotype 6 TCRβ**	ATGAGAGTTAGGCTCATCTCTGCTGTGGTGCTGTGTTTCCTAGGAACAGGCCTTGTGGACATGAAAGTAACCCAGATGCCAAGATACCTGATCAAAAGAATGGGAGAG<br>AATGTTTTGCTGGAATGTGGACAGGACATGAGCCATGAAACAATGTACTGGTATCGACAAGACCCTGGTCTGGGGCTACAGCTGATTTATATCTCATACGATGTTGATA<br>GTAACAGCGAAGGAGACATCCCTAAAGGATACAGGGTCTCACGGAAGAAGCGGGAGCATTTCTCCCTGATTCTGGATTCTGCTAAAACAAACCAGACATCTGTGTACTT<br>CTGTGCTAGCAGTTTACCACGTCTTAGTGCAGAAACGCTGTATTTTGGCTCAGGAACCAGACTGACTGTTCTCGAGGATCTGAGAAATGTGACTCCACCCAAGGTCTCCT<br>TGTTTGAGCCATCAAAAGCAGAGATTGCAAACAAACAAAAGGCTACCCTCGTGTGCTTGGCCAGGGGCTTCTTCCCTGACCACGTGGAGCTGAGCTGGTGGGTGAATG<br>GCAAGGAGGTCCACAGTGGGGTCAGCACGGACCCTCAGGCCTACAAGGAGAGCAATTATAGCTACTGCCTGAGCAGCCGCCTGAGGGTCTCTGCTACCTTCTGGCACA<br>ATCCTCGAAACCACTTCCGCTGCCAAGTGCAGTTCCATGGGCTTTCAGAGGAGGACAAGTGGCCAGAGGGCTCACCCAAACCTGTCACACAGAACATCAGTGCAGAGG<br>CCTGGGGCCGAGCAGACTGTGGAATCACTTCAGCATCCTATCATCAGGGGGTTCTGTCTGCAACCATCCTCTATGAGATCCTACTGGGGAAGGCCACCCTATATGCTGTG<br>CTGGTCAGTGGCCTGGTGCTGATGGCTATGGTCAAGAAAAAAAATTCCTGA

Supplementary Table 4: Experiments and GEMs depicted in Figures

Experiments	GEMs	Cell origin	CD8 depletion	Hashtag	Sorting strategy	Pooling ratio	Figures
Experiment 1	GEM 1	WT thymus	Yes	TotalSeq-C0301	iNKT cells	8.33%	Figures 2-4
		WT thymus	Yes	TotalSeq-C0302	iNKT cells	8.33%	
		WT thymus	Yes	TotalSeq-C0303	iNKT cells	8.33%	
		MHC-T thymus	Yes	TotalSeq-C0304	PILT-enriched population	25%	Figures 1-4
		MHC-T thymus	Yes	TotalSeq-C0305	PILT-enriched population	25%	
		MHC-T thymus	Yes	TotalSeq-C0306	PILT-enriched population	25%	
Experiment 2	GEM 2	WT thymus	Yes	TotalSeq-C0301	iNKT cells	8.33%	Figures 2-4
		WT thymus	Yes	TotalSeq-C0302	iNKT cells	8.33%	
		WT thymus	Yes	TotalSeq-C0303	iNKT cells	8.33%	
		MHC-T thymus	Yes	TotalSeq-C0304	PILT-enriched population	25%	Figures 1-4
		MHC-T thymus	Yes	TotalSeq-C0305	PILT-enriched population	25%	
		MHC-T thymus	Yes	TotalSeq-C0306	PILT-enriched population	25%	
	GEM 3	WT thymus	No	TotalSeq-C0301	iNKT cells	8.33%	Figures 2-4
		WT thymus	No	TotalSeq-C0302	iNKT cells	8.33%	
		WT thymus	No	TotalSeq-C0303	iNKT cells	8.33%	
		MHC-T thymus	No	TotalSeq-C0304	PILT-enriched population	25%	Figures 1-4
		MHC-T thymus	No	TotalSeq-C0305	PILT-enriched population	25%	
		MHC-T thymus	No	TotalSeq-C0306	PILT-enriched population	25%	
Experiment 3	GEM 4	Cd1d-/- thymus	No	TotalSeq-C0301	PILT-enriched population	33.30%	Figures 1-4
		Cd1d-/- thymus	No	TotalSeq-C0302	PILT-enriched population	33.30%	
		Cd1d-/- thymus	No	TotalSeq-C0303	PILT-enriched population	33.30%	
Experiment 4	GEM 5	Thymic organoid week1	No	TotalSeq-C0301	PILT-enriched population	19%	Figure 7
		Thymic organoid week2	No	TotalSeq-C0302	PILT-enriched population	20%	
		Thymic organoid week3	No	TotalSeq-C0303	PILT-enriched population	20%	
		Thymic organoid week4	No	TotalSeq-C0304	PILT-enriched population	20%	
		Thymic organoid week5	No	TotalSeq-C0305	PILT-enriched population	20%	
		Thymic organoid week1	No	TotalSeq-C0301	iNKT	0.20%	
		Thymic organoid week2	No	TotalSeq-C0302	iNKT	0.20%	
		Thymic organoid week3	No	TotalSeq-C0303	iNKT	0.20%	
		Thymic organoid week4	No	TotalSeq-C0304	iNKT	0.20%	
		Thymic organoid week5	No	TotalSeq-C0305	iNKT	0.20%	
Experiment 5	GEM 6	WT thymus	No	TotalSeq-C0301	DN thymocytes	16.60%	Figure 6
		Thymic organoid week1	No	TotalSeq-C0302	All live cells	16.60%	
		Thymic organoid week2	No	TotalSeq-C0303	All live cells	16.60%	
		Thymic organoid week3	No	TotalSeq-C0304	All live cells	16.60%	
		Thymic organoid week4	No	TotalSeq-C0305	All live cells	16.60%	
		Thymic organoid week5	No	TotalSeq-C0306	All live cells	16.60%	
