# Supplementary material for: Developmental features and unique characteristics of peptide-specific PLZF+ innate-like T cells in mice
Source: Nat Commun. 2025 Jun 6;16:5274. doi: 10.1038/s41467-025-60617-4 (PMC12144119; doi:10.1038/s41467-025-60617-4)
Supplement: Supplementary file 2 — Reporting Summary [file 41467_2025_60617_MOESM2_ESM.pdf]

nature portfolio

Corresponding author(s): Dr. Hristo Georgiev

Last updated by author(s): May 16, 2025

Reporting Summary

Nature Portfolio wishes to improve the reproducibility of the work that we publish. This form provides structure for consistency and transparency in reporting. For further information on Nature Portfolio policies, see our Editorial Policies and the Editorial Policy Checklist.

Statistics

For all statistical analyses, confirm that the following items are present in the figure legend, table legend, main text, or Methods section.

n/a	Confirmed	
[ ]	[x]	The exact sample size (*n*) for each experimental group/condition, given as a discrete number and unit of measurement
[ ]	[x]	A statement on whether measurements were taken from distinct samples or whether the same sample was measured repeatedly
[ ]	[x]	The statistical test(s) used AND whether they are one- or two-sided<br>*Only common tests should be described solely by name; describe more complex techniques in the Methods section.*
[x]	[ ]	A description of all covariates tested
[x]	[ ]	A description of any assumptions or corrections, such as tests of normality and adjustment for multiple comparisons
[ ]	[x]	A full description of the statistical parameters including central tendency (e.g. means) or other basic estimates (e.g. regression coefficient) AND variation (e.g. standard deviation) or associated estimates of uncertainty (e.g. confidence intervals)
[ ]	[x]	For null hypothesis testing, the test statistic (e.g. *F*, *t*, *r*) with confidence intervals, effect sizes, degrees of freedom and *P* value noted<br>*Give P values as exact values whenever suitable.*
[x]	[ ]	For Bayesian analysis, information on the choice of priors and Markov chain Monte Carlo settings
[x]	[ ]	For hierarchical and complex designs, identification of the appropriate level for tests and full reporting of outcomes
[x]	[ ]	Estimates of effect sizes (e.g. Cohen's *d*, Pearson's *r*), indicating how they were calculated

Our web collection on statistics for biologists contains articles on many of the points above.

Software and code

Policy information about availability of computer code

Data collection	Cytek SpectroFlo® version 3.3.0 was used to aquire flow cytometry data
Data analysis	flow cytometry data was analysed using FlowJo 10.8.1<br><br>scRNA-seq daa was analysed using:<br>Cell Ranger (v7.0.1)<br>Seurat (v4.3.0.1)<br>djvdj (v0.1.0)<br>SoupX (v1.6.2)<br>scVI (v1.0.0)<br>Nebulosa (v1.0.1)<br>UCell (v2.4.0)<br>Monocle3 (v1.3.1)<br>ggplot2 (v3.4.3)<br>SCpubr (v2.0.1)<br>Adobe Illustrator (v15.1.0 and v29.3)<br>Inkscape (v1.3.2)

For manuscripts utilizing custom algorithms or software that are central to the research but not yet described in published literature, software must be made available to editors and reviewers. We strongly encourage code deposition in a community repository (e.g. GitHub). See the Nature Portfolio guidelines for submitting code & software for further information.

Data

Policy information about availability of data

All manuscripts must include a data availability statement. This statement should provide the following information, where applicable:

- Accession codes, unique identifiers, or web links for publicly available datasets
- A description of any restrictions on data availability
- For clinical datasets or third party data, please ensure that the statement adheres to our policy

The data that support the findings of this study are provided either in supplementary figures or in the Source data file. The raw data and cellranger outputs generated in this study have been deposited in the GEO repository under accession code GSE279513 . Source data are provided with this paper.

Research involving human participants, their data, or biological material

Policy information about studies with human participants or human data. See also policy information about sex, gender (identity/presentation), and sexual orientation and race, ethnicity and racism.

Reporting on sex and gender	No human participants and biological materials are involved in this study.
Reporting on race, ethnicity, or other socially relevant groupings	No human participants and biological materials are involved in this study.-
Population characteristics	No human participants and biological materials are involved in this study.
Recruitment	No human participantsand biological materials are involved in this study.
Ethics oversight	No human participants and biological materials are involved in this study.

Note that full information on the approval of the study protocol must also be provided in the manuscript.

Field-specific reporting

Please select the one below that is the best fit for your research. If you are not sure, read the appropriate sections before making your selection.

☒ Life sciences ☐ Behavioural & social sciences ☐ Ecological, evolutionary & environmental sciences

For a reference copy of the document with all sections, see nature.com/documents/nr-reporting-summary-flat.pdf

Life sciences study design

All studies must disclose on these points even when the disclosure is negative.

Sample size	No sample size calculation was performed in this study. For RNA-seq data, sample sizes were chosen based on the number of reads needed to achieve sufficient sequencing depth. For other experiments at least 2 independent experiments with 2 biological replicates each were conducted.
Data exclusions	we removed the ambient RNA counts using SoupX (v1.6.2) and cells with high mitochondrial gene percentage. Cells with MAIT TCR or no TCR information were also removed from the analysis. Hashtag demultiplexing and doublet removal were done using HTODemux function from Seurat. Trav, Trbv and H2 genes were removed from the datasets so as not to influence downstream analysis.
Replication	Reproducibility was confirmed by repeat experiments. All repeated experiments yielded similar results. The number of experiments performed can be found in the figure legends.
Randomization	Randomization was not relevant to this study. There was no experimental grouping in this study.
Blinding	Blinding was not relevant to this study. There was no group allocation in this study.

Reporting for specific materials, systems and methods

We require information from authors about some types of materials, experimental systems and methods used in many studies. Here, indicate whether each material, system or method listed is relevant to your study. If you are not sure if a list item applies to your research, read the appropriate section before selecting a response.

Materials & experimental systems

n/a	Involved in the study	
	x	Antibodies
	x	Eukaryotic cell lines
x		Palaeontology and archaeology
	x	Animals and other organisms
x		Clinical data
x		Dual use research of concern
x		Plants

Methods

n/a	Involved in the study	
x		ChIP-seq
	x	Flow cytometry
x		MRI-based neuroimaging

Antibodies

Antibodies used

Antigen Fluorochrome Clone Vendor Cat# Dilution
CD19 Alexa Fluor® 488 B4 BioLegend 115521 1:100
MAIT TCR Alexa Fluor® 488 NA NIH tetramer core facility NA 1:400
TCR γ/δ chain Alexa Fluor® 488 GL3 BioLegend 118128 1:100
iNKT TCR PE NA NIH tetramer core facility NA 1:400
TCR β chain PE/Cyanine7 H57-597 BioLegend 109222 1:100
CD44 eFluor™ 450 IM7 Invitrogen 48-0441-82 1:200
PD-1 Super Bright™ 702 J43 Invitrogen 67-9985-82 1:100
NA Zombie Green™ Fixable Viability Kit NA BioLegend 423112 1:400

CD4 Brilliant Ultra Violet™ 395 GK1.5 BD Bioscience 115521 1:100
TCR β chain Brilliant Ultra Violet™ 496 H57-597 BD Bioscience 749915 1:100
CD8a Brilliant Ultra Violet™ 737 53-6.7 BD Bioscience 612759 1:400
NK1.1 Brilliant Ultra Violet™ 805 PK136 BD Bioscience 612759 1:100
iNKT TCR Brilliant Violet 421™ NA NIH tetramer core facility NA 1:100
NA Zombie Aqua™ Fixable Viability Kit NA BioLegend 423102 1:100
CD19 Brilliant Violet 605™ 6D5 BioLegend 115540 1:100
CD44 Brilliant Violet 650™ IM7 BioLegend 103049 1:100
CD138 Brilliant Violet 786™ 281-2 BD Bioscience 740880 1:100
TCR γ/δ chain FITC GL3 BioLegend 118106 1:100
ROR gamma (t) PerCP-eFluor™ 710 B2D Invitrogen 46-6981-82 1:800
CD24 PE M1/69 BioLegend 101808 1:100
T-bet PE/Dazzle™ 594 4B10 BioLegend 644828 1:100
PD-1 PE/Cyanine7 J43 Invitrogen 25-9985-82 1:100
PLZF Alexa Fluor® 647 R17-809 BD Bioscience 563490 1:100
CD3 APC/Fire™ 810 17A2 BioLegend 100268 1:100
PLZF Alexa Fluor® 488 Mags.21F7 BD Bioscience 563490 1:100
TCR γ/δ chain Alexa Fluor® 647 GL3 BioLegend 118134 1:100
F4/80 FITC REA126 Miltenyi 130-102-327 1:100
mouse IgG1 k isotype control Alexa Fluor® 647 MOPC-31C BD Bioscience 563490 1:100
MAIT TCR PE NA NIH tetramer core facility NA 1:400
CD19 Brilliant Violet 510 1D3 BD Bioscience 562956 1:100

DLL4 APC HMD4-1 BioLegend 130813 1:100
DLL1 PE HMD1-1 BioLegend 128307 1:100
H-2Kb APC AF6-88.5.5.3 BioLegend 116518 1:100
H-2Ld/H-2Db PE 28-14-8 BioLegend 114507 1:100

CD8a Biotin 53-6.7 Invitrogen 13-0081-86 1:100
CD4 Biotin GK1.5 BioLegend 100404 1:100
CD3 Biotin 17A2 BioLegend 100244 1:100
TCR γ/δ chain Biotin GL3 BD Bioscience 553176 1:100
CD3e PE 145-2C11 BD Bioscience 553063 1:100
CD4 PE RM4-5 BioLegend 100512 1:100
CD8a PE 53-6.7 BioLegend 100708 1:100
TCR γ/δ chain PE GL3 BioLegend 118108 1:100
NA Streptavidin, R-Phycoerythrin Conjugate NA Invitrogen S21388 1:100
CD19 Alexa Fluor® 488 B4 BioLegend 115521 1:100
NA Zombie Green™ Fixable Viability Kit NA BioLegend 423112 1:400
CD3e APC/Cyanine7 145-2C11 BioLegend 100330 1:100
CD8a APC QA17A07 BioLegend 100712 1:100
CD4 Brilliant Violet 711™ RM4-5 BioLegend 100550 1:100

CD44 Brilliant Violet 650™ IM7 BioLegend 103049 1:200
CD25 eFluor™ 450 PC61.5 Invitrogen 48-0251-82 1:100

CD4 Brilliant Ultra Violet™ 395 GK1.5 BD Bioscience 115521 1:100
TCR β chain Brilliant Ultra Violet™ 496 H57-597 BD Bioscience 749915 1:100
CD8a Brilliant Ultra Violet™ 737 53-6.7 BD Bioscience 612759 1:400
NK1.1 Brilliant Ultra Violet™ 805 PK136 BD Bioscience 612759 1:100
iNKT TCR Brilliant Violet 421™ NA NIH tetramer core facility NA 1:100
NA Zombie Aqua™ Fixable Viability Kit NA BioLegend 423102 1:100
CD19 Brilliant Violet 605™ 6D5 BioLegend 115540 1:100
CD44 Brilliant Violet 650™ IM7 BioLegend 103049 1:100
Thy1.1 Brilliant Violet 785™ OX-7 BioLegend 202553 1:100
T-bet eFluor™ 660 4B10 Invitrogen 50-5825-82 1:100
TCR γ/δ chain FITC GL3 BioLegend 118106 1:100
ROR gamma (t) PerCP-eFluor™ 710 B2D Invitrogen 46-6981-82 1:800
PLZF PE R17-809 BD Bioscience 564850 1:100
CD3 APC/Fire™ 810 17A2 BioLegend 100268 1:100

TotalSeq™-C anti-mouse Hashtag 1 NA M1/42; 30-F11 BioLegend 155861 1:100
TotalSeq™-C anti-mouse Hashtag 2 NA M1/42; 30-F11 BioLegend 155863 1:100
TotalSeq™-C anti-mouse Hashtag 3 NA M1/42; 30-F11 BioLegend 155865 1:100
TotalSeq™-C anti-mouse Hashtag 4 NA M1/42; 30-F11 BioLegend 155867 1:100
TotalSeq™-C anti-mouse Hashtag 5 NA M1/42; 30-F11 BioLegend 155869 1:100
TotalSeq™-C anti-mouse Hashtag 6 NA M1/42; 30-F11 BioLegend 155871 1:100
TotalSeq™-C anti-mouse TCR γ/δ chain NA GL3 BioLegend 118141 1:100
TotalSeq™-C anti-mouse CD279 (PD-1) NA RMP1-30 BioLegend 109127 1:100
TotalSeq™-C anti-mouse/human CD44 NA IM7 BioLegend 103063 1:100
TotalSeq™-C anti-mouse NK-1.1 NA PK136 BioLegend 108765 1:100
"TotalSeq-C0810 anti-mouse CD138 (Syndecan-1)" NA 281-2 BioLegend 352327 1:100

Validation

Antibodies were validated by the manufacturers and used in accordance with the manufacturers recommendations.
Biolegend: Each lot of this antibody is quality control tested by immunoflourescent staining with flow cytometrie analysis
BD: The production process underwent stringent testing and validation to assure that it generates a high-quality conjugate with consistent performance and specific binding activity.
Invitrogen: This antibody has been tested by flow cytometric analysis of stimulated mouse splenocytes

Eukaryotic cell lines

Policy information about cell lines and Sex and Gender in Research

Cell line source(s)

MS-5 cell-line was purchased from (Leibniz Institute DSMZ-German Collection of Microorganisms and Cell Cultures GmbH, Cat: ACC441). Lenti-X™ cells were purchased from (Takara Bio, Cat: 632180).

Authentication

cell line was not authenticated

Mycoplasma contamination

mycoplasma testing was not peformed

Commonly misidentified lines
(See ICLAC register)

No commonly misidentified cell line was used in this study.

Animals and other research organisms

Policy information about studies involving animals; ARRIVE guidelines recommended for reporting animal research, and Sex and Gender in Research

Laboratory animals

C57BL/6N (B6) mice were obtained from Charles River. B6.Cg-Tg(Cd4-cre)1Cwi/BfluJ (CD4Cre strain: #022071), B6.129S6-Del(3Cd1d2-Cd1d1)1Sbp/J (CD1d-/- strain: #008881), B6.129P2-B2mtm1Unc/J (B2m-/- strain: #002070) and B6.Cg-Rag2tm1.1Cgn/J (Rag2-/- strain: #008449) mice were purchased from the Jackson Laboratories. The B6.-Gt(ROSA)26Sortm1(CAG-Nlrc5)Khog (Nlrc5-stopflox MGI: #7286188) mice were kindly provided by Kristin Hogquist (University of Minnesota, Minneapolis). All mouse strains used in this study are on B6 genetic background. All animals used in this study were 8–12 weeks old at the time of analysis.

Wild animals

No wild animals were used in this study.

Reporting on sex

Female and male mice were used but sex was not considered in the study design.

Field-collected samples

No field collected samples were used in this study.

Ethics oversight	All experimental procedures were conducted in accordance with the local animal welfare regulations reviewed by the institutional review board and the Niedersächsisches Landesamt für Verbraucherschutz und Lebensmittelsicherheit (LAVES) under the permission 2022/306.

Note that full information on the approval of the study protocol must also be provided in the manuscript.

Plants

Seed stocks	No plant was used in this study.
Novel plant genotypes	No plant was used in this study.-
Authentication	No plant was used in this study.

Flow Cytometry

Plots

Confirm that:

- [x] The axis labels state the marker and fluorochrome used (e.g. CD4-FITC).
- [x] The axis scales are clearly visible. Include numbers along axes only for bottom left plot of group (a 'group' is an analysis of identical markers).
- [x] All plots are contour plots with outliers or pseudocolor plots.
- [x] A numerical value for number of cells or percentage (with statistics) is provided.

Methodology

Sample preparation	Single-cell suspensions were prepared on ice in FACS buffer (PBS/3% fetal bovine serum). All surface stainings were performed In FACS buffer on ice for 30 min. Intracellular staining for T-bet and PLZF was done using eBioscience™ FOXP3 / Transcription Factor Staining Buffer Set (Invitrogen, Cat: 00-5523-00), according to the manufacturer recommendations. CD1d tetramers loaded with PBS57 (analogue of a-galactosylceramide) and MR1 tetramers 66 loaded with 5-OP-RU were provided by the NIH Tetramer Core Facility.
Instrument	Cytek Aurora
Software	SpectroFlo® version 3.3.0<br>FlowJo 10.8.1
Cell population abundance	Cell populations were abundant enough for any of the analysis.<br>For experiment 4 cells were pooled from 3 organoids to achieve sufficient cell numbers
Gating strategy	In all experiments, debris were excluded by using Forward scater/Side scatter(FSC/SSC). Doublets were excluded by double forward (FSC-W and FSC-A)<br>Fig. 5: Dead cells were excluded with Zombie Aqua™ Fixable Viability Kit. Cells that stained negative were considere live. Then TCRγδ+ cells were excluded. From here PILT cells were identified as TCRβ+ and PLZF+ cells<br>Fig. 8:Dead cells were excluded with Zombie Aqua™ Fixable Viability Kit. Cells that stained negative were considere live. Then TCRγδ+ cells and mCherry+ were excluded. The further gating is shown in figure 8.<br>sorting: Dead cells were excluded with Zombie Aqua™ Fixable Viability Kit. Cells that stained negative were considered live. Then CD19+ B cells and MR1-tet 5-OP-RU+ MAIT cells were excluded. After removal of CD1d-tet PBS-57+ iNKT cells, the PILT enriched poulation was identified as CD44+ and/or PD-1+ cells.

- [x] Tick this box to confirm that a figure exemplifying the gating strategy is provided in the Supplementary Information.
